# A critical initialization for biological neural networks

**DOI:** 10.1038/s41586-026-10528-1

**Published:** 2026-05-20

**Authors:** Marius Pachitariu, Lin Zhong, Alexa Gracias, Amanda Minisi, Crystall Lopez, Carsen Stringer

**Affiliations:** HHMI Janelia Research Campus, Ashburn, VA USA

**Keywords:** Network models, Cellular neuroscience

## Abstract

Intrinsically generated, brainwide neural activity displays macroscopic coordination among large populations of neurons that persists beyond the biophysical timescales of individual neurons^1–3^. It is not well understood how these macroscopic behaviours arise from microscopic, short-lived interactions between pairs of neurons. Here we show that the eigenvalue spectrum and dynamical properties of large-scale neural recordings in mice are similar to those produced by linear dynamics governed by a random symmetric matrix that is critically normalized. An exception was population activity in hippocampal area CA1, which resembled an efficient, uncorrelated neural code that may be optimized for information storage capacity. High-dimensional, global activity modes emerged in critically normalized artificial networks and persisted under sparse, clustered or spatial connectivity. These dynamics were useful for solving time-dependent tasks such as a zero-shot working memory task.

## Abstract (web summary)

Basic properties of symmetric random matrices can explain the emergence of macroscopic patterns in neural networks, suggesting that initialization conditions of connected neural populations may confer computational advantages across the mouse brain.

## Main

Intrinsically generated neuronal activity contains macroscopic modes of coordination between neurons that extend across the entire mouse brain^2–5^. More activity variance is concentrated into the top dimensions of neural activity than would be expected for independently firing neurons. At the same time, there is no low-dimensional cutoff of variance concentration, and variance scales as a power-law of the eigenmode number^2^. As yet, there are no mechanistic models that can explain this scaling of variance, but macroscopic variability in general has been hypothesized to arise from neural network dynamics operating in either a critical or chaotic regime^6–11^.

Emergent macroscopic structure has also been studied in artificial neural networks, usually in the context of neural network initialization. Good initializations can directly satisfy the temporal requirements of many computational tasks^12–14^, or at least substantially accelerate subsequent learning and lead to better final models^15–17^. Commonly used initializations scale the amplitudes of the weight matrix by the inverse square root of the number of in-units, out-units or a combination of these^15,18^. More complex initialization schemes are rarely tested (but see refs. ^19–21^). A better understanding of emergent macroscopic structure may lead to better initialization schemes for modern, complex models such as transformers^22^, state space models^23^ and deep signal processing models^24^.

Coordinated, brainwide neural activity has been observed across several timescales, including seconds-long patterns^2,25^. Such persistent activity has been hypothesized to form the basis for working memory^26^, but it is not well understood how the long timescales of working memory can emerge from individual neurons with fast dynamical properties. We follow previous work to assume that the interactions in a high-dimensional neural network can be approximated by random matrices^27–31^, which can summarize the combined effect of a large number of interactions in the network. We use basic properties of symmetric random matrices such as the semicircle law^32^ to explain the emergence of macroscopic patterns in neural networks, and show that these dynamics match recorded neural datasets in a quantitative way.

## Random matrix dynamics

**Fig. 1:**
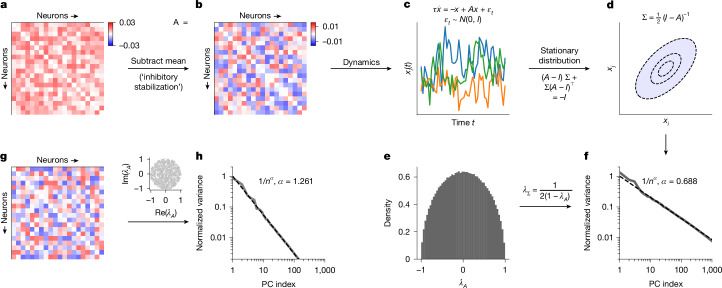
Dynamical system with random connectivity produces power-law covariance structure. **a**, Symmetric connectivity matrix *A*, with entries drawn from a uniform random distribution. **b**, Connectivity matrix from **a** with the mean of the matrix subtracted, representing global inhibition. **c**, Example neural activities from dynamical system with connectivity *A*. **d**, Covariance matrix Σ derived from the Lyapunov equation for the stationary distribution. **e**, Eigenvalues of random symmetric matrix *A* follow the semicircle law^32^. **f**, Sorted eigenvalues of covariance matrix Σ follow a power-law. **g**, Same as **a**, but connectivity now is non-symmetric. Inset, Eigenvalues of random non-symmetric matrix *A* follow the circular law^39^. **h**, Eigenvalues of covariance matrix Σ follow a power-law with a faster decay than the symmetric case.

**Extended Data Fig. 1:**
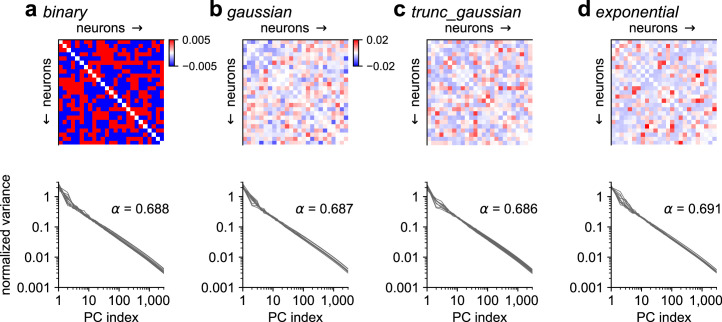
Simulations of dynamics from connectivity matrices with different probability distributions. **a**–**d** Top: connectivity matrices, in which independent connections are drawn from various distribution: Bernoulli, Gaussian, truncated Gaussian and exponential. In all cases, the mean of the distribution is subtracted off, and values are scaled to a spectral radius of 0.998. Bottom: Eigenspectra of covariance matrices from 10 simulations with random instantiations of the connectivity matrix, average *α* reported across the simulations.

Our initial modelling goal was to reproduce the power-law scaling of variance across modes of neural population activity^2^. We make the simplifying assumption that, during spontaneous activity, the non-linear network dynamics can be approximated as linear dynamics around a stationary point. In addition, we assume that each model unit (which could be a neuron or a group of neurons) generates independent stochastic variation, such as may be observed for example in the Poisson-like firing to external stimuli. When the stochastic inputs are Gaussian, this model describes a stochastic Ornstein–Uhlenbeck process^10,33–36^, though this assumption is not required for the results below. All that is required is independence across units and across time. The interaction matrix *A* contains independent random, positive numbers distributed uniformly, representing the excitatory interactions between units (Fig. 1a). To stabilize the dynamics, we subtract the mean of this matrix, which in the brain could be implemented with global inhibitory feedback (Fig. 1b and Methods). The results below still hold when the interactions are drawn from other distributions (Extended Data Fig. 1).

**Extended Data Fig. 2:**
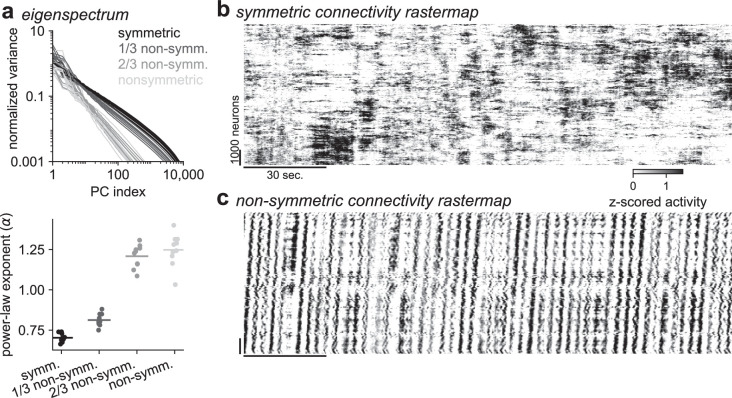
Simulations of dynamics from connectivity matrices with varying dynamics. **a**, Eigenspectra of covariance matrices from 10 simulations with connectivity defined by random symmetric matrices, random nonsymmetric matrices, and matrices with partial symmetry (1/3, 2/3) (top), and power-law exponents of the eigenspectra, lines denote mean (n = 10) (bottom). **b**, Rastermap of activity from an example simulation from a random symmetric connectivity matrix. **c**, Same as **b** for a random non-symmetric matrix.

When the interaction matrix *A* is symmetric, the covariance of the resulting multi-dimensional activity can be calculated directly from *A* using the Lyapunov equation^37^, and its eigenvalues can be related to the eigenvalues of *A* (Fig. 1c,d). We further scale *A* to have a spectral radius (largest eigenvalue) of 1 or close to 1 (Methods). We call such matrices critically normalized. In this case, it can be shown both mathematically and numerically that the eigenvalues of the covariance decay as a power-law with exponent of approximately 2/3 (Fig. 1e,f and Extended Data Fig. 2).

The case for non-symmetric interactions *A* proceeds similarly and results in a power-law of variances with approximate exponent 1.25 (Fig. 1g,h and Extended Data Fig. 2). We could not obtain a direct mathematical estimate in this case and leave this as an open problem. When the interaction matrix is only partially symmetric, the eigenvalues decay as a power-law with intermediate exponent between 2/3 and 1.25 (Extended Data Fig. 2). Thus, critically normalized matrices with varying degrees of symmetry can model the covariance spectrum of a variety of high-dimensional real-world datasets, which we have observed previously to follow power-law decays with varying exponents^38^. Here we focus exclusively on the spontaneous activity observed in large-scale neural recordings from the mouse brain, and leave the modelling of other data for future work.

**Extended Data Fig. 3:**
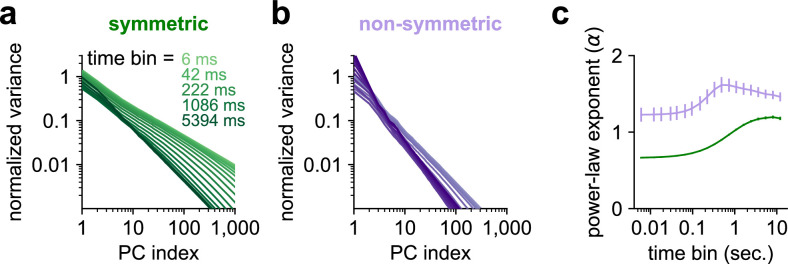
Effect of bin size on power-law exponent in simulations. **a**, Distribution of variance across principal components for simulations binned at different bin sizes (starting at 6 ms), using random symmetric, critically-normalized connection matrices, averaged over 10 simulations. **b**, Same as **a** for non-symmetric connectivity matrices. **c**, Summary of fitted power-law exponents as a function of bin size for the simulations in **a** and **b**. Data are presented as mean values +/− SEM (n = 10 simulations).

We note that some of our modelling assumptions are similar to those in ref. ^10^, but our quantitative predictions (around 2/3 power-law for symmetric matrices) are very different from that study (around 4/3 power-law). These are quite different predictions: in a 2/3 power-law for a population of 10,000 neurons, approximately 1,500 dimensions are required to account for 50% of the variance, whereas in a 4/3 power-law, 3 dimensions are sufficient. The discrepancy is due to the theory in ref. ^10^ being developed for very large binning windows, which may account for the slow timescales of neural activity but not the faster timescales that may be more relevant to neural computations. We found empirically that windows as large as 10 s are needed to reach a power-law close to 4/3 in the simulations (Extended Data Fig. 3).

**Extended Data Fig. 4:**
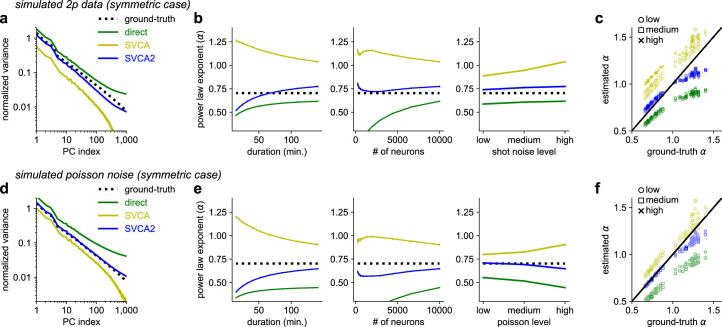
Effect of noise, recording duration and number of neurons on eigenvalue estimation from simulations and data. **a**, Estimation of eigenvalue spectra in simulations with realistic, challenging noise conditions with Poisson single-neuron noise, followed by convolution with a GCaMP-like kernel, added shot noise, and deconvolution (n=10 simulations each). Multiple estimation methods shown: SVCA, SVCA2 or direct SVD. **b**, Estimation of the eigenvalue spectrum across a range of manipulations in simulations. Data are presented as mean values +/– SEM (n=10 simulations). **c**, Estimating the eigenvalue spectrum for simulations with different ground truth exponents and different noise levels *α*. **def**, Same as **abc** for simulations that contain the Poisson single-neuron noise but lack the two-photon imaging noise. This is meant to replicate the electrophysiological data.

Finally, we used simulations to benchmark three empirical methods for estimating eigenvalue spectra in noisy data: direct eigendecomposition, shared variance component analysis (SVCA) and a new method called SVCA2. In realistic simulations, which reproduced the type of noise in either two-photon (2p) or electrophysiological (ephys) recordings, we found that SVCA2 was closest to the ground-truth power-law exponent (Extended Data Fig. 4), so we used SVCA2 below.

## Intrinsic structure in neural recordings

**Fig. 2:**
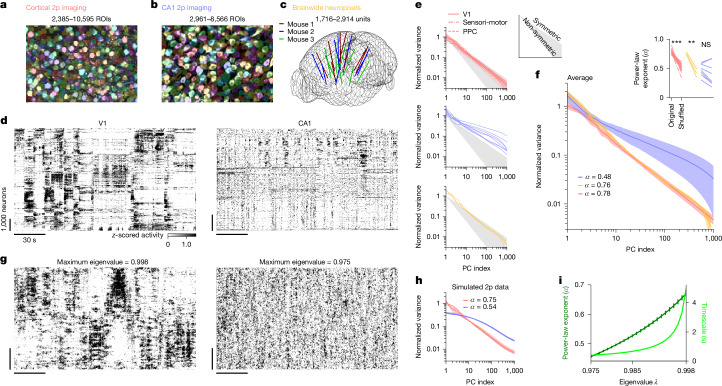
Power-law correlation structure in neural recordings. **a**, Two-photon calcium imaging in mouse cortex, at 22 Hz. Maximum projection image overlaid with units found in example V1 recording (out of 18 cortical recordings). **b**, Same as **a**, from hippocampal CA1, at 22 Hz (out of eight CA1 recordings). **c**, Eight-probe Neuropixels recordings^60^. **d**, Rastermaps^38^ of neurons from example V1 recording (left) and example CA1 recording (right). **e**, Eigenvalue spectrum from each recording. Shaded area bounded by power-law exponents from simulations with symmetric and non-symmetric matrices. **f**, Eigenvalue spectra across recordings with power-law fits. Data are presented as mean values ± s.d. Inset, power-law exponents compared with temporal shuffling. Two-sided paired *t*-test, *P* values for cortical recordings (*n* = 18), neuropixels (*n* = 3) and CA1 (*n* = 8) are 4.5 × 10^−10^, 0.009 and 0.07, respectively. **g**, Example rastermaps of simulated symmetric dynamics with near-critical normalization (left) and non-critical normalization (right) (out of ten simulations of each). **h**, Eigenvalue spectra estimated from simulations as in **g** (*n* = 10 simulations). **i**, Scaling of maximum eigenvalue with the power-law exponent, and with the longest timescale in simulations. Data are presented as mean values ± s.e.m. (*n* = 10 simulations). NS, non-significant. Schematic in **c** reproduced with permission from ref. ^2^, American Association for the Advancement of Science.

Previous work estimated the power-law decay of spontaneous population activity and obtained exponents of 1–1.2 (refs. ^2,38^), which would be in the middle of the range between symmetric and non-symmetric connectivity in the model. However, these estimates were upper bounds, because they were obtained with SVCA, which is biased substantially upward (Extended Data Fig. 4). Furthermore, these estimates were made in either 1.2-s or 0.3-s bins in transgenic mice expressing GCaMP6s—a sensor that attenuates higher frequencies in the neural activity. To obtain better estimates of the eigenvalue spectrum of the neural activity in small bins, we recorded at around 22 Hz from cortex in transgenic jGCaMP8s mice and in mice with jGCaMP8s injections (2,385–10,344 regions of interest (ROIs)), thus taking advantage of the much faster dynamics of jGCaMP8s (Fig. 2a). In addition, we used SVCA2 (Methods). We also performed the eigenvalue estimation on large-scale recordings from hippocampal area CA1 in GCaMP6f transgenic mice and in mice with jGCaMP8s injections, all recorded at 22 Hz (2,961–8,566 ROIs; Fig. 2b). Finally, we ran the same analyses on brainwide neural recordings with eight simultaneous Neuropixels probes (1,716–2,914 single-units; Fig. 2c), which we also binned at 22 Hz (ref. ^2^). In all cases, mice were head-fixed in complete darkness, and performed spontaneous behaviours, and for all analyses based on 2p calcium imaging, we used spike-deconvolved data (Methods).

**Extended Data Fig. 5:**
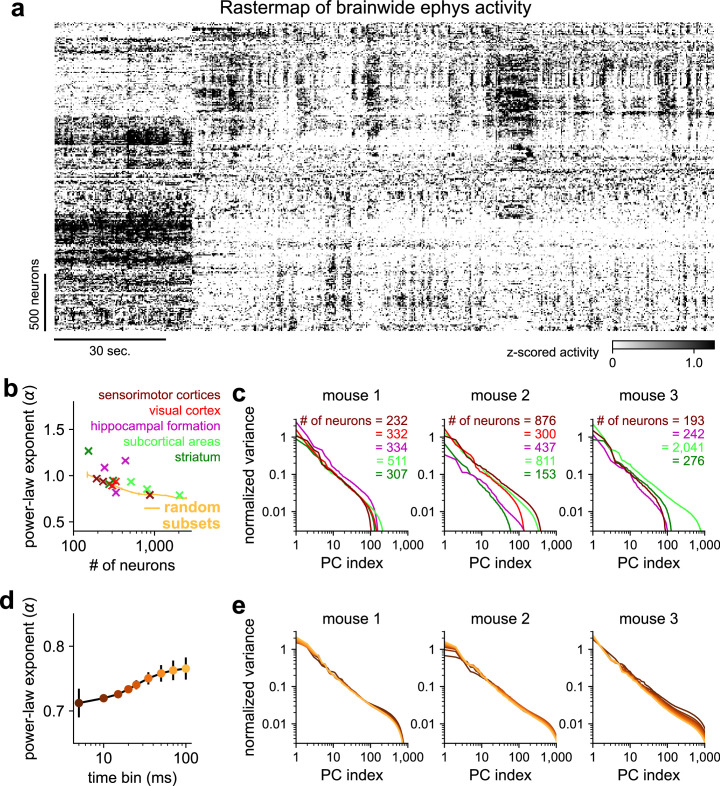
Effect of area splitting and binning on electrophysiological recordings. **a**, Rastermap of brainwide ephys recording, binned at 7Hz for plotting. **b**, Power-law exponents fit to eigenvalues estimated using SVCA2, as a function of the number of neurons after splitting into brain areas, or with random subsets of neurons. **c**, Eigenvalue curves used to compute the exponents in **b**. **d**, Power-law exponent as a function of bin size. Data are presented as mean values +/– SEM (n=3 recordings). **e**, Eigenvalue curves as the bin size is varied.

**Extended Data Fig. 6:**
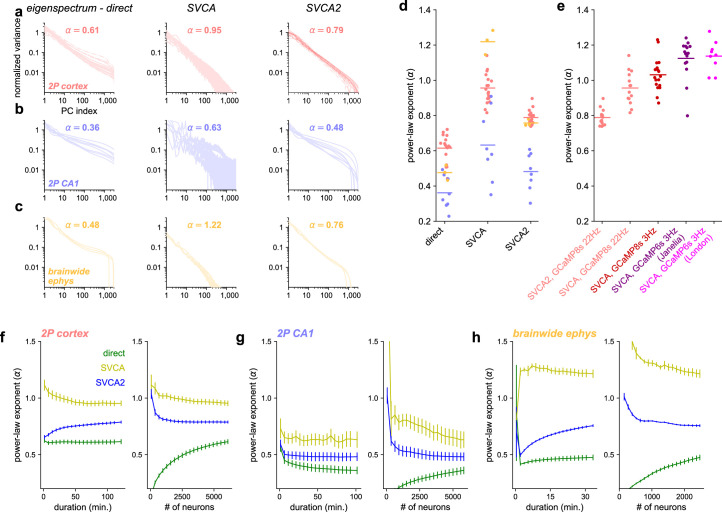
Eigenvalue estimation with different methods and on different recordings. **a**, Direct, SVCA and SVCA2 estimation for the 2p cortex data. **b**, Same as **a** for the 2p CA1 data. **c**, Same as **b** for the brainwide electrophysiological data. **d**, Power-law exponents estimated from linear fits to **abc**. **e**, Power-law exponents for cortical 2p data compared to different binning (22Hz vs 3.14Hz) and compared to GCaMP6s recordings recorded at ~3Hz on different microscopes. **f**, Power-law exponent as a function of duration and number of neurons for real data (2p cortex). **g**, Same as **f** for 2p CA1 data. **h**, Same as **f** for brainwide electrophysiological data.

We first visualized the population recordings using Rastermap—a method for reordering neurons in a raster plot so that neurons with similar activity patterns are placed next to each other^38^. Qualitatively, the cortical 2p recordings (Fig. 2d, left) and the brainwide ephys recordings (Extended Data Fig. 5a) displayed macroscopic coordination in neural firing, which was mostly absent in the CA1 recordings (Fig. 2d, right). The variance spectra of this activity decayed with power-law exponents in the range of 0.7–0.85 for both the cortical 2p recordings and the brainwide ephys recordings, close to the estimates expected from the stochastic dynamics of a random symmetric matrix (Fig. 2e). These exponents were reduced substantially after shuffling in time the activity of individual neurons (Fig. 2f) and were stable with respect to the number of neurons recorded and duration of recordings (Extended Data Fig. 6). Similar to the simulations, other eigenvalue estimation methods were biased substantially relative to SVCA2 (Extended Data Fig. 6d,e and Extended Data Fig. 4).

Estimating the power-law exponents for different brain areas in the ephys data did not reveal large differences (Extended Data Fig. 5b,c), and varying the time binning did not substantially change the power-law exponent (Extended Data Fig. 5d,e). There were also no clear differences between V1, posterior parietal cortex and sensorimotor cortex in the two-photon data (Fig. 2e). However, the hippocampal CA1 population activity had a variance spectrum that decayed much slower, with exponents in the range of 0.4–0.5, that were not changed substantially by single-neuron shuffling (Fig. 2e,f). The macroscopic, long timescale dynamics in the cortical and brainwide data resembled those in simulations that were critically normalized (Fig. 2g, left). This required normalizing the largest eigenvalue to be very nearly 1, as an incompletely normalized interaction matrix did not result in long timescale macroscopic structure, similar to the CA1 spontaneous activity (Fig. 2g–i).

**Fig. 3:**
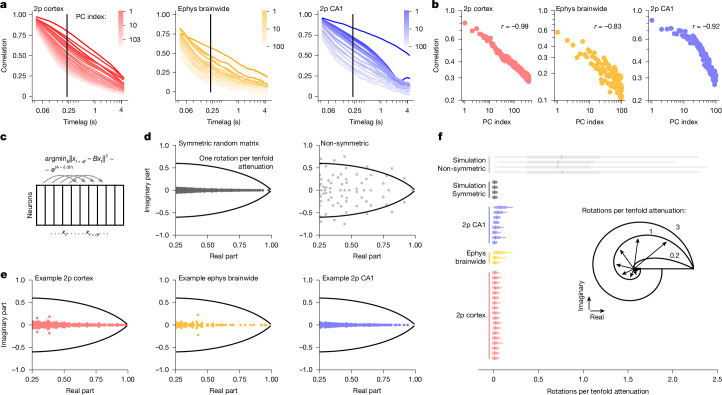
Dynamical properties of neural macroscopic dynamics. **a**, Estimated ACGs (noise-normalized) of the neural PCs, averaged across all recordings of the same type (*n* = 18 2p cortex; *n* = 3 ephys; *n* = 8 2p CA1). **b**, Average auto-correlation for each PC at a time lag of 5 bins (around 0.23 s) across all recordings, Pearson’s *r* in log–log scale. **c**, Schematic of dynamic mode decomposition. **d**, Eigenvalues of the estimated DMD matrices at a time lag of around 0.23 s for simulations with symmetric (left) and non-symmetric (right) random matrices. **e**, Same as **d** for example real recordings. **f**, Estimated number of rotations per tenfold attenuation for the complex eigenvalues estimated from DMD, shown per recording and simulation. This estimation is performed on eigenvalues with a real part greater than 0.25; numbers varied from 80 to 991 across datasets. Thick and thin lines indicate 25–75% and 5–95% ranges, respectively, with the median shown as a darker line.

The model also predicts that the variance of the principal components (PCs) should covary with their intrinsic timescales (Methods). To verify the prediction in the data, we computed the auto-correlograms (ACGs) of the PCs of each recording (Fig. 3a). We took care to estimate the ACGs in a manner that takes the noise level of each component into account (Methods). In addition, we smoothed the ephys recordings in time to find the eigenvectors, but reordered these based on their raw, non-smoothed variance. This step was necessary due to the relatively fewer neurons and much shorter recording times in the ephys data, both of which increase the estimation noise of the eigenvectors. Across all recordings, we found that PCs with more variance had slower temporal dynamics, as predicted by theory (Fig. 3b).

Next we investigated a key difference between symmetric and non-symmetric dynamics: the complexity of the eigenvalues of their dynamics. Symmetric matrices have real eigenvalues, whereas non-symmetric random matrices have eigenvalues distributed on a disk in the complex plane^39^. Symmetric, stable matrices produce relaxation dynamics, whereas non-symmetric random matrices produce substantial rotational dynamics. The Rastermap visualization gave us the first indication that spontaneous activity does not contain rotational dynamics because there was no consistent drift or sequential activity across the neural subpopulations (Fig. 2d and Extended Data Fig. 5a). To quantify this effect, we turned to time-lagged dynamic mode decomposition (DMD) (Fig. 3c)—a popular method for identifying dynamical effects in sequential data^40,41^. For linear dynamical systems, the eigenvalues of the DMD matrix are related directly to the eigenvalues of the linear dynamics matrix. Furthermore, the eigenvalues of the DMD matrix for symmetric random dynamics have nearly zero complex parts, unlike those from non-symmetric random dynamics (Fig. 3d). Estimated from the data, the eigenvalues of the DMD matrices all had near-zero complex parts (Fig. 3e). We quantified this with the number of full rotations per tenfold attenuation of the magnitude of the complex eigenvector projection (Fig. 3f). Only the simulated, non-symmetric dynamics reached levels of rotation that affect the dynamics substantially.

**Extended Data Fig. 7:**
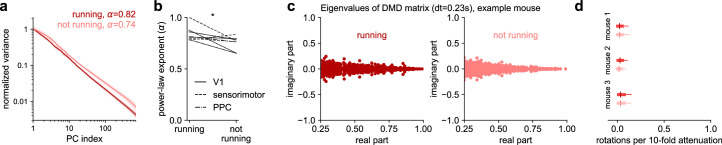
Dynamical properties in running vs not running states. **a**, For mice that spent at least 13% of the time running and at most 75%, data was divided into subsets based on running and eigenvalue spectra were estimated. **b**, Summary of power-law exponent changes paired across mice. **c**, Eigenvalues of DMD matrix for example mouse in running and not running state (like Fig. 3e). **c**, Summary of changes to rotational components of eigenvalues with magnitude at least 0.25 (like Fig. 3f), number of components varied from 237 to 375. Only three sessions contained sufficiently long uninterrupted bouts of running to perform this analysis.

**Extended Data Fig. 8:**
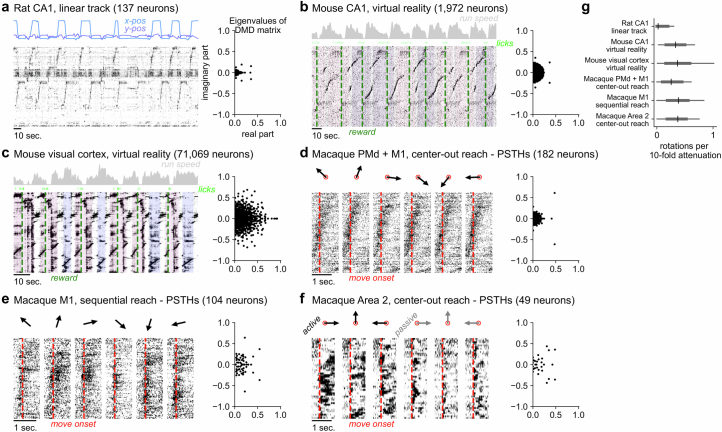
Eigenvalue estimation on noisy simulations with varying number of neurons and timepoints. Each panel shows: (left) a Rastermap of an example recording segment, and (right) the eigenvalues obtained from an application of the DMD method from Fig. 3. **a**, CA1 recording with place cells in a freely moving rat on a linear track from^87^. **b**, CA1 recording with place cells in virtual reality in a mouse from^89^. **c**, Mouse visual cortex recording in virtual reality from^91^. **d**, Macaque recording from PMd and M1 averaged over trials, from^94^. Six example reaches shown with corresponding PSTHs. **e**, Macaque M1 recording binned by reach angle and averaged over trials from^96^. Six example reach directions are shown. **f**, Macaque area 2 recordings averaged over trials^98^. **g**, Estimated number of rotations per 10-fold attenuation for the complex eigenvalues estimated from DMD, shown per recording. This estimation is performed on eigenvalues with a real part greater than 0.1, numbers varied from 11-463 across recordings. Thick and thin lines indicate 25–75% and 5–95% ranges respectively, with the median shown as a darker line.

In some cases, we were able to split the recordings into running and not running timepoints. The power-law exponents and DMD eigenvalue distributions seemed very similar between the two behavioural states, with a small but significant increase in power-law exponent (Extended Data Fig. 7). In other recordings where sequential activity is expected, for example, in response to external, behaviourally relevant cues, we found eigenvalues from DMD with significant rotational components (Extended Data Fig. 8). We suspect that the rotational components observed in these recordings are related, at least in part, to the structure of the task and environment.

## Dynamics under structured connectivity

So far, we have found the neural data to match the structure of dynamics with a critically normalized, symmetric random matrix. This model, however, ignores many structural properties of real neuronal circuits. For example, connections between neurons are sparse, they depend on distance in tissue, and they also depend on cell type. In this section we show that if at least a small fraction of the connections are global then global activity modes still emerge, with the same power-law distribution of eigenvalues. We focus on symmetric matrices.

**Fig. 4:**
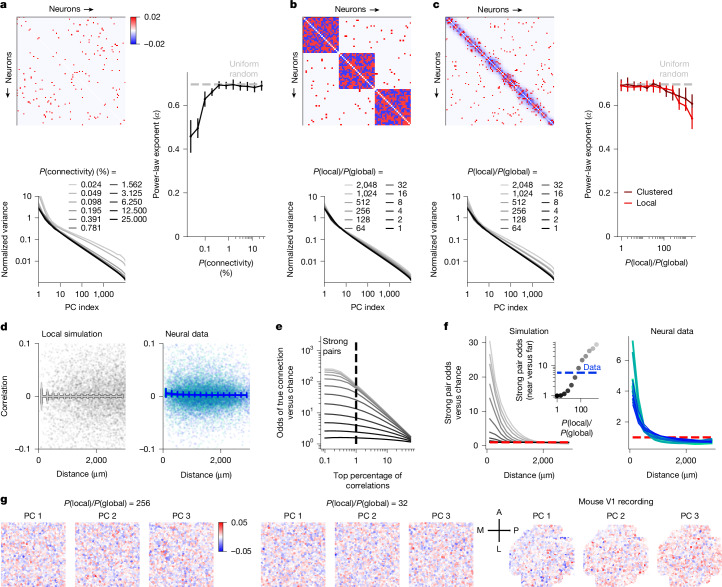
Persistence of global activity modes under biophysical connectivity patterns. **a**–**c**, Top, Connectivity matrices with symmetric sparse (**a**), clustered (**b**) and localized (**c**) weights. Inhibition is set in proportion to the probability of connection for each weight. Bottom, variance decay of PCs for simulations with different levels of sparsity (**a**), clustering (**b**) and localization (**c**). Right, Power-law exponents for these conditions. Data are presented as mean values ± s.d. (*n* = 10 simulations per condition). Dashed line, the 0.69 value from Fig. 1. **d**, Scatterplot of pairwise correlations versus distance in either a simulation with local connectivity (left) or mesoscopic, single-unit recordings from ref. ^43^ (right). Solid lines, mean for each simulation/recording; error bars, s.d. across neuron pairs. **e**, Strong pairs (top 1% correlated pairs) are likely to be true connections across a wide range of sparse connectivity as in **a**. **f**, Dependence of strong pair odds with distance in either simulations with local connectivity (left) or recordings (right), red line denotes odds of one. Inset, monotonic relation between the strong pair odds at near versus far distances (which can also be estimated from data) and the true ratio of local and global probability of connection. **g**, Top PCs are global in both recordings and simulations, even for very large bias towards local connectivity. A, anterior; L, lateral; M, medial; P, posterior.

Sparse, symmetric random matrices also follow the Wigner semicircle law when the sparsity is not very high^42^. Thus, the dynamics of the resulting systems retain the same 2/3 power-law scaling of variance across activity modes. Empirically, we found that the connection probability needed to be at least 0.4% in a network of 10,000 units to leave the variance spectrum unchanged (Fig. 4a). Similar properties apply to clustered and spatially structured stochastic connectivity, as long as each connection is drawn from a mean zero distribution (Methods). In simulations, the variance spectrum remained unchanged as long as the global connection probability was at least 1% of the local probability (Fig. 4b,c).

We investigated the spatial model further, as we could potentially match it to properties of spatially sampled neural recordings from a previous study^43^. The pairwise correlations across neurons in the simulation did not have significant correlation with distance, similar to the data (Fig. 4d). Thus, average correlations cannot be used to infer the strength or length scale of spatial connectivity. We hypothesized that we could use instead the highest correlated pairs of neurons as a better match to connectivity. We define as ‘strong pairs’ those pairs of units or neurons in the top 1% highest correlation. In simulations, the strong pairs had a much higher chance to be connected compared with chance (Fig. 4e). The odds of a given pair being a strong pair varied strongly with distance, with a similar length scale as that used to simulate the data (Fig. 4f). Further, the odds ratio of strong pairs between near pairs and far pairs was proportional to the ground-truth probability ratio of local and global connections (Fig. 4f). We observed a similar relation with distance of strong pair likelihoods in the data^44^ (Fig. 4f). Thus, strong pairs are enriched at short distances, and that may be a reflection of a higher connection probability. Finally, we note that, in both data and in simulations with strong local connectivity, the top PCs are always global (Fig. 4g).

## Computations with symmetric dynamics

**Fig. 5:**
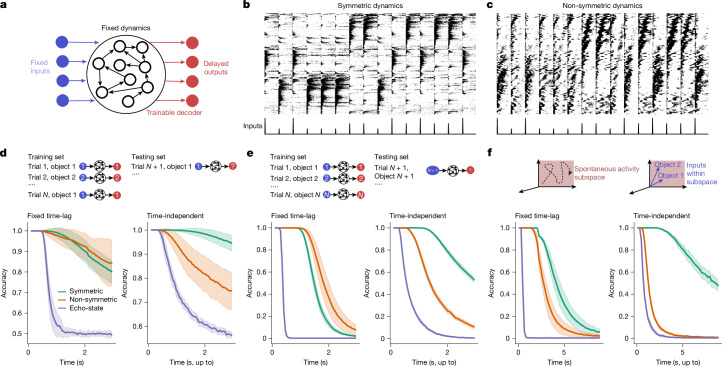
Working memory with symmetric linear dynamics. **a**, Model of persistent activity with fixed inputs, fixed linear dynamics (as in Fig. 1) coupled with a trainable linear readout. **b**, Example simulated neural activity with three distinct inputs and symmetric dynamics. **c**, Same as **b** with non-symmetric dynamics. **d**, Benchmarking working memory retrieval by training and testing the decoder on two inputs, with different noise instantiations for train and test trials. Top, schematic of benchmark; bottom left, retrieval accuracy at a fixed time lag for symmetric and non-symmetric models, as well as for echo-state networks; bottom right, same as bottom left for time-independent decoding at all time lags up to a maximum. **e**, Same as **d** for a zero-shot working memory task in which test and train inputs are different, in which retrieval requires recalling the input features. **f**, Same as **e** but with input patterns drawn from the subspace of spontaneous activity. Data are presented as mean values ± s.d. (*n* = 20 simulations).

Next we explored the computational properties of linear dynamics with random, symmetric and critically normalized connectivity. We focused on working memory tasks, which are building blocks for more complex computations. For simplicity, we assumed that the dynamics of the network are fixed and random, that the input patterns are also fixed and random (Fig. 5a) and that spontaneous activity continues uninterrupted during tasks and stimulus presentations as observed typically in mice^2,4^, thus inducing trial-to-trial variability and decoder noise. Persistent activity in the network can be read out at some temporal delay (Fig. 5a), and we assume the readout is a simple decoder that can be optimized on a training set, for example, using Hebbian learning rules. We compare several critically normalized forms of dynamics: linear symmetric, linear non-symmetric and non-linear non-symmetric. The latter are generally referred to as echo-state networks^12^, liquid state machines^13^ or reservoir computing^45,46^.

We consider three different task scenarios. First, a simple delayed binary classification task that is similar to typical working memory experiments in animals (Fig. 5d). This task requires long timescales but does not require large memory capacity. Second, we consider a delayed, zero-shot working memory task with 1,000 training inputs and 1,000 testing inputs, which requires recalling the features of an arbitrary random input, a task that humans can execute. This task requires long timescales, large memory capacity and flexibility to encode arbitrary new patterns (Fig. 5e). Third, we consider the same zero-shot working memory task, but allowing for the inputs to be represented by macroscopic patterns of spontaneous activity—a hypothetical mechanism for enhancing working memory duration (Fig. 5f). Across these tasks, we found that both symmetric and non-symmetric linear dynamics performed well at time lags of up to several seconds, whereas echo-state networks struggled to maintain more than half a second of memory, probably due to their chaotic dynamics^47^ not being robust to noise. Moving to a time-independent version of the same task, we found that symmetric dynamics performed substantially better than non-symmetric dynamics (Fig. 5d–f). To understand this difference, note that long timescales in non-symmetric models are almost always associated with large imaginary eigenvalues (Fig. 3d), and thus substantial rotational dynamics (Fig. 5c and Extended Data Fig. 2c). Thus, a stable representation cannot be maintained across time, even though the information is present in the network. Finally, we note that allowing the recurrent connections to change by means of optimization would probably improve performance for all these networks.

## Discussion

The modelling results we presented are quite general. They hold for arbitrary distributions of the independent noise, with nearly arbitrary distributions of connection strengths, and when the connectivity matrix has low-rank or spatial structure. Across all these modelling choices, the substantial difference between symmetric and non-symmetric systems holds (2/3 versus around 1.25 power-law exponent), which corresponds to a dramatic difference in effective dimensionality. The 0.75–0.8 power-law exponent in the neural recordings may therefore indicate that a higher-dimensional code is preferable for neural computations, and that it is achieved through critically normalized, symmetric interactions. Symmetric interactions are widespread in the brain: brain areas^48^ and pairs of neurons^49^ are often reciprocally connected. The critical normalization needed to generate macroscopic dynamics can be achieved in a self-tuned way as suggested previously in other contexts^50^. For example, an initially unstable system can be scaled down, by pruning or rescaling connections, until it is stable^51^, through various mechanisms^52,53^.

Models with random connectivity and non-linear dynamics have a long history in computational modelling^12,13,45^. These modelling efforts typically exploit the (near-) chaotic dynamics in such networks to perform non-linear computations that require memory. Recent deep learning methods show that even models with linear dynamics can perform such tasks as long as they are deep^23,54^. Thus, we may hypothesize that spontaneous activity in the mouse brain reflects the initialization of a brainwide neural network that can provide ideal conditions for computations that require dynamics and memory. There already exists evidence that this scaffold is used to represent motor states^2^, and that laboratory tasks trigger a brainwide cascade of neural activity similar to the patterns observed in spontaneous activity^4,55–57^. Perhaps all the learning that needs to happen in such tasks is on the readout or feedforward connections from sensory inputs to the brainwide dynamical reservoir. This could explain why spontaneous activity reflects a randomly initialized network state, rather than a modified state with a different distribution of eigenvalues. Alternatively, a different subset of dynamics might ‘turn on’ during task execution^58^. Distinguishing between these scenarios will require large-scale neural recordings with longitudinal tracking during the learning and execution of memory-guided tasks—an increasingly more feasible experiment as large-scale recording techniques are refined^59^.

## Methods

All experimental procedures were conducted according to the Institutional Animal Care and Use Committee (IACUC) at Howard Hughes Medical Institute (HHMI) Janelia. Data analysis and simulations were performed in Python using pytorch and numpy, and figures were made using matplotlib and jupyter-notebooks^61–65^.

### Data acquisition

#### Animals

All experimental procedures were conducted according to IACUC ethics approval received from the IACUC board at HHMI Janelia Research Campus. We performed 18 recordings in cortex in: (1) 12 mice bred to express jGCaMP8s^66^ in excitatory neurons: TetO-jGCaMP8s × Camk2a-tTA mice (available as JAX 037717 and JAX 007004); (2) 3 mice bred to express jGCaMP8s^66^ in the somas of excitatory neurons: riboL1–jGCaMP8s × Slc17a7-Cre (similar to JAX 039267 without IRES; and JAX 037512) and (3) 3 mice bred to express tdTomato in inter-neurons VGAT-CRE × Ai14 (JAX 016962; JAX 007914) with injections of a dual virus Thy1s:TTA (AAV9, 1.64 × 10^13^ vector genomes ml^−1^) and TRE3G:RiboL1–jGCaMP8s (AAV9, 2.45 × 10^13^ vector genomes ml^−1^) as in ref. ^67^, see also ref. ^68^. We also performed eight recordings in hippocampal CA1 in six mice bred to express GCaMP6f in excitatory neurons: Thy1-GCaMP6f GP5.17 mice (JAX 025393)^69^, as well as in two wild-type C57 mice (JAX 000664) with injections of the same RiboL1-jGCaMP8s virus combination described above. These mice were male and female, and ranged from 2 to 12 months of age. Mice were housed in reverse light cycle, and were pair-housed with their siblings before and after surgery. Holding rooms are set to a temperature of 70 °F ± 2 °F, and humidity of 50% relative humidity ± 20%.

#### Surgical procedures

Surgeries were performed in adult mice (P35–P125) following procedures outlined in refs. ^70,71^. In brief, mice were anaesthetized with isoflurane while a craniotomy was performed. Marcaine (no more than 8 mg kg^−1^) was injected subcutaneously beneath the incision area, and warmed fluids plus 5% dextrose and buprenorphine 0.1 mg kg^−1^ (systemic analgesic) were administered subcutaneously along with dexamethasone 7 mg kg^−1^ by the intramuscular route. In the canula implants, the same total dexamethasone dose was administered tapered over 3 days: 4 mg kg^−1^ on the first day, 2 mg kg^−1^ on the second day and 1 mg kg^−1^ on the third day.

For the visual cortical windows (which included the posterior parietal cortex), measurements were taken to determine the bregma–lambda distance and location of a 4-mm circular window over the V1 cortex, as far lateral and caudal as possible without compromising the stability of the implant. For the sensorimotor windows, the craniotomy was centred at −0.75 mm anteroposterior (AP) and 2.2 mm mediolateral (ML) from bregma. A 4- and 5-mm double window was placed into the craniotomy so that the 4-mm window replaced the previously removed bone piece and the 5-mm window lay over the edge of the bone. For the hippocampal windows, the craniotomy was centred at 1.8 mm AP and 2.0 mm ML from bregma. Cortex was aspirated and a 3-mm glass coverslip attached to a stainless-steel was implanted over the dorsal CA1 region. CA1 surgeries were similar to those described in ref. ^71^.

After surgery, ketoprofen (5 mg kg^−1^) was administered subcutaneously and the animal allowed to recover on heat. The mice were monitored for pain or distress and ketoprofen 5 mg kg^−1^ was administered for 2 days following surgery.

#### Imaging acquisition

We used a custom-built 2p mesoscope^72^ to record neural activity, and ScanImage^73^ for data acquisition. We used a custom online *Z*-correction module (now in ScanImage), to correct for *Z* and *XY* drift online during the recording. As described in ref. ^70^, for the visual area and hippocampal recordings, we used an upgrade of the mesoscope that allowed us to approximately double the number of recorded neurons using temporal multiplexing^74^.

The mice were free to run on a styrofoam cylinder. Mice were acclimatized to running on the ball for several sessions before imaging, and one mouse was trained on a virtual reality task for 2 weeks before the recording. The field of view was selected such that large numbers of neurons could be observed, with clear calcium transients. Recordings were performed for 100–150 min at a rate of 22 Hz. We performed one recording session in each of the 26 mice, and did not perform a sample size analysis. Blinding and randomization were not used. Recordings from refs. ^43,75^ were acquired at a rate of 3 Hz. Recordings from refs. ^2,76^ were acquired at a rate of 2.5–3 Hz, on a Thorlabs Bergamo microscope.

#### Processing of calcium imaging data

Calcium imaging data were processed using the Suite2p toolbox (v.0.9.3)^77^, available at www.github.com/MouseLand/suite2p. Suite2p performs motion correction, ROI detection, neuropil correction and spike deconvolution as described elsewhere^2^. We used a neuropil subtraction coefficient of 1.0. For the 22-Hz recordings, we used all ROIs output by Suite2p above a signal-to-noise ratio (SNR) threshold of 0.3, which included dendritic processes, to increase the number of units recorded. The SNR for the activity trace *x* for each ROI was defined as

$$\,{\rm{S}}{\rm{N}}{\rm{R}}=1-\frac{{\rm{V}}{\rm{a}}{\rm{r}}[{x}_{t}-{x}_{t-1}]}{2\,{\rm{V}}{\rm{a}}{\rm{r}}[{x}_{t}]}$$

(similar to ref. ^78^). 61 ± 16% (mean ± s.d.) ROIs had an SNR greater than 0.3, resulting in a range of 3,981–10,595 ROIs across recordings.

We improved the spike deconvolution here by using the latest version of Suite2p, which will be described in an upcoming manuscript. Our approach was similar to that in ref. ^78^, where a neural network is used to predict the ground-truth spikes from the noisy convolved traces. Unlike ref. ^78^ we trained the model on a large number of simulations single-neuron spiking activity convolved with GCaMP-like dynamics and we used a Unet predictive model^79^ with a style-vector to capture temporal context independently for each deconvolved trace^80^. We verified that the deconvolved traces better capture neural activity on real data with less noise by evaluating the responses to visual stimuli presented at known times. To estimate the effect of binning in the GCaMP8s recordings (Extended Data Fig. 6e), we binned the fluorescence traces by a factor of 7 and then performed deconvolution.

#### Neuropixel recordings and processing

As described in ref. ^2^, eight-neuropixel electrode arrays were used to record simultaneously from up to 3,000 neurons across the brain in three mice^60^. On the day of recording, mice were anesthetized briefly with isoflurane while eight small craniotomies were made with a dental drill. After several hours of recovery, mice were head-fixed in the International Brain Laboratory (IBL) task setup: seated in a plastic tube with their forepaws on a wheel, surrounded by three computer screens in a light-isolated enclosure^55,81^. The electrodes were advanced slowly (approximately 10 μm s^−1^) to their final depth (4 mm or 5 mm deep), and allowed to settle for around 15 min before recording. During the spontaneous part of the recording, the computer screens were black. Data were pre-processed by re-referencing to the common median across all channels^82^. The probe locations were determined using https://github.com/cortex-lab/allenCCF, and the brain mesh in Fig. 2c was plotted using this tool, based on Allen Common Coordinate Framework data^83,84^.

These recordings were re-processed with Kilosort4 (v.4.0.14), with default settings^85^. The Kilosort4 spike sorter found 1,756, 2,837 and 2,962 neurons defined as ‘good’, with a refractory violation rate of less than 0.2 (default), from the three recordings. We excluded neurons with a firing rate of less than 0.01 Hz during the spontaneous recording period, resulting in 1,716, 2,787 and 2,914 neurons in total in each recording. The detected neurons were located across cortex, the hippocampal formation, the striatum and other subcortical areas. For the grouping in Extended Data Fig. 5b,c, the ‘visual cortex’ group consisted of primary and higher-order visual cortices; the ‘sensorimotor cortex’ group consisted of motor cortex and somatosensory cortex; the ‘striatum’ group consisted of the caudate putamen and lateral septal cortex and the ‘subcortical areas’ consisted of superior colliculus, thalamus and midbrain. Mice 1 and 2 had detected neurons in all five groups; mouse 3 had detected neurons in all groups except visual cortex. The spikes were binned at a rate of 22 Hz—the same acquisition rate as the calcium imaging. We also analysed bin sizes from 5 ms to 100 ms in Extended Data Fig. 5d,e. The period of spontaneous activity in each recording was 22–42 min long (only this period was used for analyses).

### Data analysis

We normalized the neural activity to avoid fitting single-neuron statistics. We *z*-scored the activity of each neuron so that the mean activity of each neuron is 0 and its s.d. is 1. We ran Rastermap on the recordings with 100 clusters and 128 PCs, and visualized the sorted activity using 20 neurons per bin^38^. The behavioural running state was estimated by interpolating the running trace to the recording frames, smoothing with a 25 frame Gaussian kernel, and setting a threshold of running/not running at 1/100 of the maximum smoothed running speed. We included sessions for analysis in which the mouse was running at least 10% of the time, and for the DMD analysis in which the mouse was running for at least 30 min (Extended Data Fig. 7).

#### Eigenspectrum estimation

We estimated the eigenvalues using the covariance between two halves of neurons from the recording. This is to avoid contaminating the eigenspectrum with single-neuron noise produced from the recording methods and from Poisson variability. We divided the recordings in half spatially, using a checkerboard of size 50 μm for the calcium imaging recordings, and using sections of 40 μm (eight contacts) on each Neuropixels probe in the electrophysiological recordings. We did not split the data into training and testing timepoints, as we found this inflated the power-law exponent estimates^2^ (Extended Data Fig. 4).

We estimated the power-law exponent of the eigenspectrum decay using a weighted linear regression in log space from rank 10 to 500, with weights as the inverse of the log of the rank^86^. The eigenvalue spectra are normalized by the value of the linear regression fit at rank 1. If the length of the spectrum was less than 500 due to limited numbers of neurons, then we estimated the spectrum using 10 to half the length of the spectrum.

We subsampled the number of neurons randomly in Extended Data Fig. 6f–h, and subsampled by brain region in the ephys data in Extended Data Fig. 5b,c. We subsampled timepoints in the recordings using chunks of length 50 to 4,000, spaced in time by 4,000 time points.

#### Estimating PC timescales from data

To estimate the timescales of the PCs, we must take into account the noise. A naive estimation of the ACGs would find that later PCs have smaller time-lagged correlations, but that could be due simply to these PCs having lower SNR overall, and thus all their time-lagged correlations would be lower. Instead we take a similar approach to that from SVCA: we split the data into two random subsets of neurons using a checkerboard grid with size 50 μm, and we calculate the components with a singular value decomposition (SVD) on the covariance between the two subsets of neurons. The resulting left and right singular vectors were used to project test data, and the correlograms were computed between the projections of one set of neurons and the other. The resulting estimates of the PC ACGs were then normalized to 1 at a time lag 0.

#### Estimating rotational components from data

When the dynamics matrix *A* is not symmetric, its eigenvalues are complex and the covariance of the multi-dimensional data is no longer related directly to these eigenvalues. Thus, to evaluate the complexity or rotational aspect of the dynamics directly, we cannot rely on the PCs alone. Instead, we fit linear predictive models that predict the neural population vector at time *t* + *dt* from the population vector at time *t*. Such models are referred to typically as DMD, with the small modification that we use a time lag *dt* = 0.23 s instead of the more common *dt* = 1 time sample. The reason to use a time lag is to make estimation of the rotational modes more robust and, in particular, to avoid the potential influence of short timescale artefacts arising from the deconvolution of the calcium imaging data.

To estimate DMD, we first reduced each dataset to 1,000 dimensions using PC analysis (PCA). We then used ridge regression with a penalty of 0.1 for the ephys and 0.01 for the 2p calcium imaging to predict *X*_*t*+*dt*_ from *X*_*t*_ in the reduced PCA space. Thus *X*_*t*+*dt*_ ≈ *BX*_*t*_, with *B* a square matrix mapping PCs to PCs. As usual, the neural activity was *z*-scored on a per neuron basis before applying PCA, but it was not re-normalized afterwards. Since PCA is an orthonormal projection, the eigenvalues of *B* are the same as would be expected in the full neuronal space, other than estimation errors. In the model, the matrix exponential describes the relation between *X*_*t*+*dt*_ and *X*_*t*_, regardless of whether *A* is symmetric or not:

$${X}_{t+{dt}}={\exp }^{(A-I){dt}/\tau }{X}_{t}.$$

Thus, the DMD matrix *B* we obtained from data is an estimate of ${\exp }^{(A-I)dt/\tau }$, at least in the simulations. Looking at the complexity of the eigenvalues of *B* can thus indicate whether the dynamics are rotational or not. Note that the eigenvalues ${\lambda }^{{\prime} }$ of ${\exp }^{(A-I)dt/\tau }$ are related to the eigenvalues *λ* of *A* by ${\lambda }^{{\prime} }={\exp }^{(\lambda -1)dt/\tau }$. Thus the higher the complex part of *λ*, the higher the complex part of ${\lambda }^{{\prime} }$. We can also now see why taking a larger *dt* is beneficial: when *dt* is very small relative to the timescale of the dynamics, the eigenvalues ${\lambda }^{{\prime} }$ approach 1, making it difficult to estimate their rotational component. The relation ${\lambda }^{{\prime} }={\exp }^{(\lambda -1)dt/\tau }$ could be inverted to obtain estimates of *λ*. However, this multi-step process is likely to contain a lot of estimation error, and we preferred instead to directly compare the estimated distributions of ${\lambda }^{{\prime} }$ from data with those from appropriately matched simulations. In particular, we compute the number *n*_rot10_ of rotations per tenfold attenuation of the complex eigenvector ${\lambda }^{{\prime} }$:

$$\begin{array}{c}\begin{array}{c}{n}_{\mathrm{rot}10}=k\cdot \mathrm{angle}\,({\lambda }^{{\prime} })\\ \,|{\lambda }^{{\prime} }{|}^{k}=0.1\end{array}\end{array}.$$

Thus:

$${n}_{\mathrm{rot}10}=\frac{\log 0.1}{\log |{\lambda }^{{\prime} }|}\cdot \mathrm{angle}\,({\lambda }^{{\prime} }).$$

We used *τ* = 20 ms in the simulations with symmetric matrices to match approximately the timescales of the data. Longer or shorter *τ* in the simulations would simply contract all estimated eigenvalues towards 0, but otherwise leave the number of rotations per tenfold attenuation unchanged.

We also estimated eigenvalues of dynamics using DMD on various recordings in which rodents and monkeys were performing tasks (Extended Data Fig. 8). In terms of rodent experiments, we analysed one session from refs. ^87,88^, in which rats ran down a linear track, and 137 neurons from CA1 were recorded using silicon probes. We analysed one session from refs. ^89,90^ in which mice were detecting a reward location in two different virtual reality corridors, and neural activity was recorded from CA1 using 2p calcium imaging at a rate of 15 Hz. We analysed one session from refs. ^91,92^ in which mice were performing a visual discrimination task in virtual reality, and neural activity was recorded from visual cortex using 2p calcium imaging at a rate of 3 Hz. For DMD analysis in each of these recordings, we used a ridge penalty of 0.1, and a time delay of 2 s. We also analysed ephys data collected from monkeys, compiled by the Neural Latents Benchmark challenge^93^. We binned the spikes in each of these recordings at a rate of 50 Hz, and ran DMD on the peri-stimulus time histograms (PSTHs) computed from each of the recordings, aligned to movement onset. In refs. ^94,95^, the monkey completed a maze task with one to many targets, with 108 different configurations—in the figure we plotted example PSTHs from single target trials. In refs. ^96,97^, the monkey performed reaches between random elements of a grid—we binned the reaches based on movement direction angle into 15 bins. In refs. ^98,99^, the monkey performed a centre-out reaching task in eight different directions (active trials), and on a subset of trials the joystick was moved (passive trials); recordings were performed in somatosensory Area 2. In each of these recordings, we performed DMD with a ridge penalty of 0.1 and a time delay of 200 ms.

#### Local correlation structure

In Fig. 4, we computed the correlations using the recording sampling rate (3 Hz). For the simulations, we used the correlation matrix derived from the eigenvectors. For all the analyses we excluded neuron pairs within 20 μm of each other. The pairwise correlations were binned in 200-μm bins (Fig. 4d).

We defined the top 1% of correlations per neuron as the ‘strong pairs’ for each neuron. We then computed the probability distribution of the strong pairs across spatial bins of 200 μm. This distribution was normalized by the distribution of all other correlations across bins, producing the strong pair odds versus chance shown in Fig. 4f. The strong pair odds, near versus far, was the ratio of the first bin of this curve (within 200 μm) to the average of the last four bins of this curve (2,200–3,000 μm).

### Dynamical systems analysis

We assume that neural activity is governed by linear dynamics with independent stochastic inputs. In the case of normally distributed inputs, this model becomes the familiar Ornstein–Uhlenbeck process, with connectivity *A*^33^:

1 $$\tau \frac{d{\bf{x}}}{{dt}}=-{\bf{x}}+A{\bf{x}}+{{\epsilon }}_{{\bf{t}}},$$

where the noise is a Wiener process.

The stationary distribution of the neural covariance matrix Σ is defined by the Lyapunov equation^37^:

$$(A-I)\Sigma +\Sigma {(A-I)}^{{\rm{\top }}}=-I.$$

When *A* is symmetric, the solution is given by

2 $$\Sigma =\frac{1}{2}{(I-A)}^{-1}.$$

For the non-symmetric case, the solution must be calculated numerically^100^ (see also ref. ^101^ for an example of solving for *A*).

For the symmetric case, we can derive the decay of the eigenvalue spectrum directly, under the assumption that *A* is a random symmetric matrix with an eigenspectrum distribution of a semicircle from −1 to 1. We assume here that the number of units is large enough to treat the eigenvalue distribution as an exact semicircle distribution and ignore finite size effects. The rank *n* of eigenvalue ${\lambda }_{A}^{n}$ of *A* is defined by the integral of the semicircle distribution density *p*(*λ*_*A*_) from ${\lambda }_{A}^{n}$ to 1. We scale *p*(*λ*_*A*_) to a maximum of 1 for convenience of the calculations. If we define *θ* as the angle subtended by $p({\lambda }_{A}^{n})$ on the semicircle, we can use geometric arguments to show that:

$$\begin{array}{c}\begin{array}{c}n=\pi \left(\frac{\theta }{2\pi }\right)-\frac{1}{2}\cos \theta \sin \theta \\ =\frac{1}{2}(\theta -\cos \theta \sin \theta )\end{array}\end{array}.$$

The eigenvalues of the covariance Σ, denoted by *λ*, are related to the eigenvalues *λ*^*A*^ of the connectivity matrix *A*:

$$\lambda =\frac{1}{2(1-{\lambda }_{A})}\Rightarrow \,{\lambda }_{A}=1-\frac{1}{2\lambda }.$$

Thus, we have

$$\theta ={\cos }^{-1}({\lambda }_{A})={\cos }^{-1}\left(1-\frac{1}{2\lambda }\right).$$

Plugging this into the equation for rank *n* and using $\sin (\theta )=\sqrt{1-{\cos }^{2}(\theta )}$ we have:

$$\begin{array}{l}2n={\cos }^{-1}\left(1-\frac{1}{2\lambda }\right)-\left(1-\frac{1}{2\lambda }\right)\sqrt{1-{\left(1-\frac{1}{2\lambda }\right)}^{2}}\\ \,=\,{\cos }^{-1}\left(1-\frac{1}{2\lambda }\right)-\left(1-\frac{1}{2\lambda }\right)\sqrt{\frac{1}{2\lambda }}\sqrt{2-\frac{1}{2\lambda }}.\end{array}$$

We next used Wolfram Alpha^102^ to obtain the Taylor expansion for $\sqrt{2-x}$ and Puiseux expansion for ${\cos }^{-1}(1-x)$ where *x* is small. Keeping only terms in which *x* is raised to a power less than 2, gives

$$\begin{array}{l}2n\approx \sqrt{\frac{1}{\lambda }}+\frac{1}{24{\lambda }^{3/2}}-\sqrt{\frac{1}{\lambda }}+\frac{5}{8{\lambda }^{3/2}}+{\mathcal{O}}\left(\frac{1}{{\lambda }^{5/2}}\right)\\ \,\approx \frac{2}{3{\lambda }^{3/2}}\,\Rightarrow \lambda \propto \frac{1}{{n}^{2/3}}.\end{array}$$

Thus, the eigenvalues decay approximately as a power-law with exponent 2/3, which is very close to the value we found in simulations.

As mentioned in the text, this is different from the value of 4/3 found in ref. ^10^; the discrepancy is due to estimating the ‘long time window covariance’, which assumes that the data are binned in infinitely long windows. The authors of ref. ^10^ argue that the formula converges for short windows (greater than 50 ms), which seems to use the single-neuron timescales as a reference. However, when the dynamical systems are close to critical, the emergent timescales are much longer, and thus a much longer window is needed to reach the stable state. Thus, the derivation in ref. ^10^, although interesting, can capture only the infraslow timescales of neural activity and predicts a 4/3 power-law on a rank plot in this case.

We note that the 4/3 exponent, while not explicitly calculated there, can be derived easily from Eq. (16) in ref. ^10^:

$$p(x)=\frac{\sqrt{2}}{\pi }{x}^{-\frac{7}{4}},$$

which integrated gives:

$${\int }_{0}^{X}p(x)dx\propto {x}^{-\frac{3}{4}},$$

which results in a power-law exponent of approximately 4/3 in a rank plot^10^.

#### Relating timescales to eigenvalues

In addition to estimating the decay of variances of the PCs, we also want to evaluate dynamical, temporal properties of the data and relate them to the model. In the model, the timescales of the system are related to the eigenvalues of *A* and therefore to the eigenvalues of Σ, following from the matrix exponential solution to the Lyapunov equation:

$$X(t)={e}^{(A-I)t/\tau }X(0)+{\int }_{0}^{t}{e}^{(I-A)(t-{t}^{{\prime} })/\tau }\sigma d{W}_{{t}^{{\prime} }}.$$

The second term on the right is a noise term that is independent of *X*(0). Using the SVD decomposition of *A* = *UΛU*^*T*^, with *Λ* having the eigenvalues ${\lambda }_{A}^{i}=1-1/(2{\lambda }_{i})$ on the diagonal, the multi-dimensional system can be divided into independent scalar equations:

$${U}_{i}^{T}X(t)={e}^{({\lambda }_{{A}^{i}}-1)t/\tau }{U}_{i}^{T}X(0)+{\rm{noise}}.$$

Thus, the PC component projections ${U}_{i}^{T}X$ have an auto-correlation that decays as

$${e}^{({\lambda }_{{A}^{i}}-1)t/\tau }={e}^{-\frac{t}{2{\lambda }_{i}\tau }},$$

and, thus, the timescales of the PCs are monotonic with the amplitude of the eigenvalue *λ*_*i*_. For the purposes of the analysis in Fig. 3, we need only observe the cross-correlation at time lag *t*, although its exact decay with *λ*_*i*_ cannot be predicted owing to the unknown single-unit timescale *τ*.

### Simulations of dynamical systems

We simulated 10,000 neurons governed by the dynamics in Eq. (1). For the dynamics simulations, we performed integration using the Euler–Maruyama method and used a step size of 2 ms and a timescale *τ* for each neuron of 20 ms using pytorch^62,103^. The random noise was drawn from a Gaussian with a mean of zero and s.d. of one for each neuron at each time step. We ran 80 simulations on a graphics processing unit in parallel, with random initial conditions drawn from a Gaussian with mean 0 and s.d. = 1, each consisting of 60,000 time steps, and discarded the first 4,000 time steps. To replicate the sampling rate in the data, we binned every 23 timepoints (approximately 22 Hz). We also *z*-scored the unit activities, as in the data. When we visualized the rastermaps of simulations, we ran 40 simulations each consisting of 100,000 time steps, to obtain longer continuous segments of dynamics.

For testing the eigenspectrum estimation methods (Extended Data Fig. 4), we normalized the activity of each neuron by its s.d., applied a relu and then used the activity of the neuron as the mean of a Poisson process, scaled by different values to represent different levels of noise. We multiplied the activity by 0.7, 0.5 and 0.3, representing ‘low’, ‘medium’ and ‘high’ noise levels respectively. To simulate 2p recordings, we used the ‘medium’ noise Poisson activity traces and convolved each trace with an exponential filter with a decay time constant of 0.25 s. All the activity traces were then scaled by a factor of eight and 400 was added to each trace, and then each trace was scaled individually by a random number drawn from an exponential distribution with 0.001 added to it, to approximate a distribution of SNR values across neurons, with a minimum SNR close to 0.3. We applied shot noise to each trace, by using the trace as the mean of a Poisson process, and then applied deconvolution as in the real neural data. The exponential distribution had mean values of 0.5, 0.2 and 0.08, representing ‘low’, ‘medium’ and ‘high’ shot-noise levels, respectively. The ‘medium’ shot-noise level had a mean SNR across recordings of approximately 0.56, similar to our neural recordings.

#### Dense connectivity

We drew the excitatory connections between neurons from a uniform random distribution from zero to two. We subtracted off the mean connectivity (one). We set the diagonal values of the matrix to zero. When this matrix is symmetric, its eigenspectrum distribution follows the semicircle law; for the non-symmetric case, it follows the circular law^104^. We divided the matrix by a scalar so that the largest real value of the eigenvalues of the matrix was 0.998, setting *A* so that it is critically normalized.

#### Sparse/varied connectivity

We created the sparse symmetric random matrices with random zero or one connections drawn from a Bernoulli with a mean varying from 2.4 × 10^−4^ to 0.25 (Fig. 4a). The mean of the Bernoulli was subtracted globally from the entire matrix, resulting in a matrix with zero mean connectivity. The diagonal of the matrix was set to zero. Random sparse symmetric matrices and graphs also follow the semicircle law, when the sparsity is not too high^42,105–107^.

We created the clustered symmetric random matrices by setting the Bernoulli mean to 0.5 for within-cluster (local) connectivity, and the mean out-of-cluster (global) connectivity in a range from 2.4 × 10^−4^ to 0.5 (Fig. 4b). This results in a ratio of the probability of local connection to the probability of global connection (*P*(local)/*P*(global)) ranging from 1 to 2,048. Each cluster consisted of 500 neurons. The mean of the Bernoulli distribution for each entry was subtracted (0.5 within-cluster, smaller outside), resulting in mean zero connectivity across the matrix. The diagonal of the matrix was set to zero.

We created the locally connected symmetric random matrices using as the Bernoulli mean an exponential decay function of Euclidean distances *d*_*ij*_ in micrometres between neurons in the simulation (Fig. 4c). The exponential function was defined as $0.5{e}^{-{d}_{ij}/250}$. The neurons were placed randomly on a torus of size 8,000 × 8,000 μm. The minimum value of the mean of the Bernoulli was set to a value from 2.4 × 10^−4^ to 0.5, resulting in a range of *P*(local)/*P*(global) from 1 to 2,048. Again, the mean of the Bernoulli was subtracted from each entry of the matrix, and the diagonal of the matrix was set to zero. To quantify the recovery of true connectivity (Fig. 4e), we compute the fraction of true positive connections with *P*% of top pairwise correlations per neuron, and then normalize by the average probability of positive connection in the simulation (chance).

As in the dense case, we divided each matrix by a scalar so that the largest eigenvalue of the matrix was 0.998. Because these connectivity matrices were symmetric, we computed the covariance matrix and the eigenspectrum directly from *A* using Eq. (2).

#### Simulations with inputs

For Fig. 5 we added external inputs to the simulations described above. All simulation parameters were kept the same, and the input magnitude of *m *= 2.5 was chosen to be comparable with the amplitude of the ongoing, internally generated activity. Inputs were kept on for 50 ms ending at simulation time 0 s and the readout was averaged over 100 ms of neural activity ending at the delay time on the *x* axis of Fig. 5d–f. For time-independent decoding, the 100-ms readout ended at a randomized time in the range between 120 ms at a minimum and the time on the *x* axis of Fig. 5d–f at a maximum. Inputs were assumed to have 100 features, which were projected to the 10,000 neurons in the simulations either with completely random weights (Fig. 5d,e), or with weights corresponding to the top 100 eigenvectors of spontaneous activity (Fig. 5f). Inputs were presented in pseudo-random order at fixed intervals of 3 s, except for the analyses in Fig. 5f where the intervals were 9 s. Mathematically:

$$\begin{array}{c}\tau \frac{d{\bf{x}}}{dt}=-{\bf{x}}+A{\bf{x}}+d{\bf{W}}+\\ \,+\,m\cdot \sum _{j}{{\bf{v}}}_{{\rm{\sigma }}(j)}{\delta }_{t\in ({t}_{j}-0.05s,{t}_{j})}\\ \,{{\bf{v}}}_{i}=B{{\bf{u}}}_{i},\,{{\bf{u}}}_{i}\in {{\bf{R}}}^{100}\\ {{\bf{y}}}_{j}({t}^{{\prime} })={\int }_{{t}_{j}+{t}^{{\prime} }-0.1s}^{{t}_{j}+{t}^{{\prime} }}{\bf{x}}(t)dt,\end{array}$$

where *d***W** is the Wiener process corresponding to the random noise simulation, **v**_*σj*_ is the random input added at time *t*_*j*_ and corresponding to a random mapping *σ*(*j*) to the range of inputs considered in each simulation. *A* is the critically normalized dynamics matrix as before and *B* is a 10,000 × 100 matrix of either random values or composed of the eigenvectors of the spontaneous activity generated in the absence of inputs. The readout ${{\bf{y}}}_{j}({t}^{{\prime} })$ for input *j* is calculated as the 100 ms integral of activity **x**(*t*) corresponding to a time offset of ${t}^{{\prime} }$ from the input time *t*_*j*_. For the echo-state network version^12^, we simply added a ReLu^62^ rectification on *x* at each Euler step of the same dynamical system. We scaled *A* to reach the edge of criticality, which in this case required an additional scaling factor of 1.4 to *A*.

The Rastermap visualizations in Fig. 5b,c were from 1,500 s of simulations with three inputs. There were two randomly drawn 100-dimensional inputs in Fig. 5d, with 1,000 repeats of each stimulus, half of which were used for training the decoder and half for testing. A one-nearest-neighbour decoder was used for this binary classification. There were 2,000 different, randomly drawn 100-dimensional inputs in Fig. 5e,f, with 1,000 inputs used for training and 1,000 used for testing. To decode the input, we fit a regression problem from the output of the network to the inputs, using ridge regression with L2 regularizer of 10. In this case, a classification was considered correct if the predicted input features were closest to the ground-truth input features, as opposed to any of the other 999 inputs used in the testing set.

### Reporting summary

Further information on research design is available in the Nature Portfolio Reporting Summary linked to this article.

## Online content

Any methods, additional references, Nature Portfolio reporting summaries, source data, extended data, supplementary information, acknowledgements, peer review information; details of author contributions and competing interests; and statements of data and code availability are available at 10.1038/s41586-026-10528-1.

## Supplementary information

Reporting Summary

Peer Review file

## Data Availability

The new neural recordings from this study are available at Figshare (10.25378/janelia.27854448)^108^. Datasets in Fig. 4 are publicly available at Figshare (10.25378/janelia.23712957)^75^. The eight-probe Neuropixels recordings were published previously^109^; we have uploaded the Kilosort4 processing of these recordings to Figshare (10.25378/janelia.27854448)^108^. The datasets in Extended Data Fig. 8 are publicly available^88,90,92,95,97,99^.

## Code availability

Code to reproduce all the analyses and figures is available at GitHub (https://github.com/mouseland/critical_init)^110^.
