# Supplementary material for: A critical initialization for biological neural networks
Source: Nature. 2026 May 20;655(8124):990–6. doi: 10.1038/s41586-026-10528-1 (PMC13391357; doi:10.1038/s41586-026-10528-1)
Supplement: Supplementary file 2 — Peer Review file [file 41586_2026_10528_MOESM2_ESM.pdf]

nature portfolio

Peer Review File

A critical initialization for biological neural networks

Corresponding Author: Dr Carsen Stringer

Any redactions in this file are there to maintain patient confidentiality, the confidentiality of unpublished data, or to remove third-party material.

This file contains all reviewer reports in order by version, followed by all author rebuttals in order by version.

Attachments originally included by the reviewers as part of their assessment can be found at the end of this file.

Version 0:

Reviewer comments:

Referee #1

(Remarks to the Author)
This paper compares the eigenspectral properties of spontaneous brain activity dynamics in mice with those generated by critically normalized random matrices. The authors provide theoretical and empirical demonstrations of power-law properties in dynamics generated by symmetric and non-symmetric random matrices, along with slope estimations of eigenspectra from these dynamical systems. By analyzing eigenspectra of spontaneous activity from primary visual cortex (2-photon calcium imaging), brain-wide neuronal activity (Neuropixels), and hippocampal CA1 (2-photon calcium imaging), they demonstrate that spectral properties of V1 and brain-wide spontaneous activity match those of critically normalized symmetric random matrices, while CA1 exhibits higher-dimensional activity.

The theoretical framework and analysis appear solid and reliable. However, I have significant concerns about the paper's framing and contextualization of results that must be addressed before publication.

Major Concerns:

1. The paper's title, abstract, and introduction emphasize connections to neural network initialization, yet this concept remains underdeveloped throughout the manuscript. The central claim linking spontaneous activity to network initialization lacks adequate justification and raises several critical questions:

- Why should spontaneous activity be considered equivalent to biological neural network initialization? Spontaneous activity generated by developed brains represent fully matured (or "trained," in artificial neural network terminology) systems, not randomly initialized networks.

- Even if spontaneous dynamics reflect linear dynamical systems with random interaction matrices, this does not establish spontaneous activity as random network initialization.
The rationale behind this conceptualization requires substantial clarification and theoretical support.

2. The abstract ends with a hypothesis that spontaneous activity reflects critical initialization optimized for learning time-dependent tasks. This claim lacks adequate support:

- What evidence supports this hypothesis? Which specific results in the paper substantiate these claims?

- If the authors believe their characterized dynamics benefit time-dependent task learning, direct evidence must be provided.

3. If critical initialization being optimal for learning represents an underlying theoretical assumption (rather than a hypothesis to be tested in this paper), it should be presented upfront with clear connections to existing literature. The paper's contribution relative to previous theoretical work requires better contextualization.

Unless the initialization connection can be substantially strengthened with direct evidence, I recommend removing it from

the paper's central claims (including the title) and treating it as a discussion point. This would make the work more exploratory, which is acceptable, but would require better contextualization with prior related work and clearer questions.

Technical Comments:

1. The removal of train/test splits in SVCA analysis, justified through simulated data with added Gaussian noise (Fig S4), requires additional validation:

- Do results depend on noise amplitude or type?

- The higher exponent estimations for noisy data shown in Fig S4 were somewhat expected: added noise may destroy higher principal components, leading eigenspectrum estimation to yield higher power-law exponents (reflecting lower-dimensional activity) for noisy versus noiseless data. Given the inherent noise in neural recordings, shouldn't SVCA account for this noise in estimations, perhaps through train/test splits? Without train/test splits, SVCA may overestimate dimensionality by failing to properly account for data noise. Can the authors elaborate more on the rationale behind their choice.

- In general, sensitivity analysis showing how conclusions depend on SVCA approach choice would strengthen this methodological decision.

2. Two factors changed from previous work (Stringer et al., Science, 2019): GCaMP6s to GCaMP8s and removal of train/test splits in SVCA. Since the main claim involves V1 spontaneous activity matching symmetric random matrix eigenspectra, the individual contribution of each change to the lower power-law exponent estimation should be demonstrated separately; either via running the same analysis on the older data collected with GCaMP6s, or redoing the same analysis in this paper with train/test split.

3. While bin size effects on exponent estimation are intuitive, several questions remain:

- How should the right bin size be determined? Which bin size would reflect the true dimensionality of activities? Wouldn't that require cross-validation?

- Can the linear dynamical system with random matrix interactions predict bin size effects on eigenspectrum estimation?

- Do predictions across different bin sizes consistently support the same conclusions regarding symmetric versus non-symmetric random matrix dynamics?

4. It would be helpful to demonstrate the sensitivity of the analysis to the number of recorded neurons. Power-law fits across different neuron numbers would establish minimum requirements for robust estimations.

5. Regarding the Neuropixels recordings:

- Which specific brain areas are included in the data?

- While V1 and brain-wide activity show similar eigenspectral properties distinct from CA1, it seems unlikely that CA1 alone among all recorded areas exhibits different spectral properties.
Breaking down brain-wide recordings into functionally similar area subgroups could address this concern, though sufficient neuron numbers per area may be limiting (relating to comment #4).

Summary:

This work presents interesting findings on neural activity spectral properties, but the connection to network initialization requires substantial strengthening or removal from central claims. The technical analysis would benefit from additional methodological validation and clearer presentation of results across brain regions.

Referee #2

(Remarks to the Author)
1. Summary of the key results

The authors measure the scaling of the variance spectra of neurons in a mouse brain, which captures how quickly the variance of data falls off with the inclusion of an increasing number of principal component subspaces. The authors compare the found variance spectra with a random neural network model with fully random weights, and with a random neural network model with random weights that are "symmetrized", showing that the symmetrized network alone matches the variance scaling found widely across the mouse brain.

The authors further compare with a data-driven model: Dynamic Mode Decomposition (DMD). This model estimates the eigenvalues of the linearized dynamics, and the authors found that these eigenvalues have small imaginary part, consistent with the symmetric linear network model.

The authors conclude that the dynamics of the mouse brain mainly lack sequential/rotational structure, and are rather much more consistent with dynamics induced by symmetric connectivity profiles.

2. Originality and significance

The work appears to be novel, including clarifying and extending existing theoretical work.

3. Data & methodology

The quality of data and validity of the collection methods appear to be high. One exception is that time binning in the case of the neuropixel data seems unnecessary and may impact the results. Is the time binning meant to capture a measure of membrane potentials or firing rates, and why? What is the downside of using the raw neuropixel data?

The results are presented in a clear way.

4. Appropriate use of statistics and treatment of uncertainties

Statistics appear to be properly treated.

5. Conclusions

We had a few comments to make on the results and conclusions:

5a. Using free probability theory applied to large matrices, we believe that the spectral properties of
$C = \int_0^\infty e^{At} e^{A^T t} dt$
(the integral solution to the continuous time Lyapunov equation) can be related to those of the symmetric random matrix $A + A^T$ as $N \to \infty$. Alternatively, one can argue that as $N \to \infty$, A and A^T become more commutative (the norm of their expected commutator shrinks to zero like 1/N). This reveals that the symmetric part of A governs the spectral scaling of C. Hence in the case of asymmetric A with the spectral radius approaching 1, one should find the same spectral scaling of C (even though the two scenarios have different asymmetric parts). Namely, in the idealized limit of $N \to \infty$ and time binning -> 0, the scaling in the case of Figure 1h and 1f should be the same.

Considering this, we recommend that the authors either:
(1) describe the flaws in our logic above if they exist, and perhaps clarify these points in the main text if appropriate
(2) if our logic above is correct, elucidate what details in the analysis result in different scalings of Figure 1h versus 1f such as time binning or finite N effects.

5b. We believe the authors are somewhat vague on the precise brain areas that are recorded from using the neuropixel probes, and this description could be improved.

5c. Many sequential processes are known to occur in the rat brain, such as hippocampal replay and brain waves of various frequencies. The authors should discuss their results in relation to these known sequential processes, and if the time binning excludes these processes it should be justified.

6. Suggested improvements

6a. The scaling exponents could easily be dependent on animal state. Since the animal is freely moving, this includes moments where the animal is still, and moments where the animal is moving. We recommend that the authors divide the dataset into sets of behaviors and analyze the scaling exponents and DMD results in these sets individually. A simple approach could be to simply divide the data into "moving" and "not moving" based on some threshold of detected movement.

6b. It is possible that the scalings reported from the neuropixel data are averages of different scalings in different brain regions. We believe it would be worth breaking the neuropixel probe data into regions based on anatomical or functional considerations, and seeing if each of these regions yield the same scaling as the whole.

6c. We think that a sanity check of the methods introduced by the authors could be helpful, perhaps in the supplementary materials. This would constitute testing their methods on either data where sequential behavior should be seen (such as motor cortex during walking) or on influential models of brain-driven sequential behavior, to show that the methods of the paper will give a positive result for asymmetry-driven dynamics in these basic cases.

6d. As stated above in point (5c), the authors should clarify the choice of time bin for the neuropixel data and the potential consequences of this, such as being too coarse to capture brain waves or replay events.

7. References

This appears to be thorough.

8. Clarity and context

The manuscript is relatively clear, excepting points we make in some of the comments above.

Referee #3

(Remarks to the Author)
I co-reviewed this manuscript with one of the reviewers who provided the listed reports.

Version 1:

Reviewer comments:

Referee #1

(Remarks to the Author)
I thank the authors for their thorough response and for addressing the majority of my previous comments. I have several remaining questions regarding the working memory task, as detailed below:

The addition of the working memory (WM) task is a valuable step toward connecting the model's spectral properties to its functional performance. However, a few key points still require clarification:

- The structure of the WM task and the specific way models are implemented to solve it remain unclear. The current schematics do not sufficiently clarify the process; for example, the meaning of the dotted arrows in the diagrams is not defined. Please provide a more detailed walkthrough of the task implementation and ensure all visual symbols in the figures are explicitly explained.

- The manuscript would benefit from a more thorough justification of the performance differences between the models:

If the symmetric model's effectiveness stems from slower spectral decay—meaning more involvement of higher-order PCs—the authors should explain why this improves performance. Since higher-order PCs typically have faster timescales (as shown in figure 3), it is not immediately intuitive why they would enhance working memory capacity. The significant underperformance of the echo-state network is also not clearly explained. A more detailed mechanistic account is needed to help the reader understand why these specific spectral and architectural properties lead to such divergent results.

- The reasoning behind utilizing three different versions of the WM task should be made more explicit. It would be helpful to clarify whether each variant is intended to probe a specific facet of network capacity or a distinct computational bottleneck.

- There is a mismatch between the caption and the figure content. Fig 2 panel (b), for example, does not reflect the eight-probe Neuropixel recordings mentioned.

(Remarks on code availability)

Referee #2

(Remarks to the Author)
Than you for carefully addressing the issues. These additions appear to largely address our concerns, with some smaller points that we lay out below.

(5c and 6c) Thanks for these additions. We believe that a word of interpretation about the results in Figure S8, perhaps alongside a summary measure such as the rotation count, would be helpful to add to the manuscript.

(6d) "Reconciling LFP oscillations with this data probably requires a different discussion, and it is not one we are well equipped to conduct in this particular manuscript with this particular kind of data."
Thank you for examining the effects of small time binning. We believe it would be helpful to include a brief discussion of possible reasons why the methods do not detect LFP oscillations, even when small time bins are used.

(Remarks on code availability)

Referee #3

(Remarks to the Author)
I co-reviewed this manuscript with one of the reviewers who provided the listed reports.

(Remarks on code availability)

Open Access This Peer Review File is licensed under a Creative Commons Attribution 4.0 International License, which permits use, sharing, adaptation, distribution and reproduction in any medium or format, as long as you give appropriate credit to the original author(s) and the source, provide a link to the Creative Commons license, and indicate if changes were made.
In cases where reviewers are anonymous, credit should be given to 'Anonymous Referee' and the source.
The images or other third party material in this Peer Review File are included in the article’s Creative Commons license, unless indicated otherwise in a credit line to the material. If material is not included in the article’s Creative Commons license and your intended use is not permitted by statutory regulation or exceeds the permitted use, you will need to obtain permission directly from the copyright holder.
To view a copy of this license, visit https://creativecommons.org/licenses/by/4.0/

Referee #1 (Remarks to the Author):

This paper compares the eigenspectral properties of spontaneous brain activity dynamics in mice with those generated by critically normalized random matrices. The authors provide theoretical and empirical demonstrations of power-law properties in dynamics generated by symmetric and non-symmetric random matrices, along with slope estimations of eigenspectra from these dynamical systems. By analyzing eigenspectra of spontaneous activity from primary visual cortex (2-photon calcium imaging), brain-wide neuronal activity (Neuropixels), and hippocampal CA1 (2-photon calcium imaging), they demonstrate that spectral properties of V1 and brain-wide spontaneous activity match those of critically normalized symmetric random matrices, while CA1 exhibits higher-dimensional activity.

The theoretical framework and analysis appear solid and reliable. However, I have significant concerns about the paper's framing and contextualization of results that must be addressed before publication.

Thanks for the review. We adjusted the framing and contextualization to align more closely to your suggestions below. At the same time, we added a new theoretical figure illustrating the computational properties of symmetric linear dynamics (new Figure 5). We also added more technical evaluations to demonstrate the accuracy of the data analysis methods (new Figures S4, S6), and further applications of the method to other publicly-available datasets, (new Figure S8). We also recorded more 2p datasets including from other cortical regions and in other behavioral states like running, which increased the number of datasets from 10 to 26 (added in Figures 2 and 3, and in new Figure S7). Finally, we added a figure showing division of electrophysiological data into brain areas (new Figure S5), but we caution against over-interpreting this.

Major Concerns:

1. The paper's title, abstract, and introduction emphasize connections to neural network initialization, yet this concept remains underdeveloped throughout the manuscript. The central claim linking spontaneous activity to network initialization lacks adequate justification and raises several critical questions:

- Why should spontaneous activity be considered equivalent to biological neural network initialization? Spontaneous activity generated by developed brains represent fully matured (or "trained," in artificial neural network terminology) systems, not randomly initialized networks.

- Even if spontaneous dynamics reflect linear dynamical systems with random interaction matrices, this does not establish spontaneous activity as random network initialization.

The rationale behind this conceptualization requires substantial clarification and theoretical support.

We had multiple sources of motivation in the original manuscript. Of these, we had chosen to emphasize the (theoretical) link to ANN initialization because the scaling of connection strengths in those networks (1/sqrt(N)) is the same scaling as in our models. Based on the reviewer feedback, we now emphasize the other sources of motivation, i.e. the need to generate macroscopic, long timescale neural activity from microscopic, short-lived interactions between pairs of neurons. We changed the first two sentences of the abstract and reorganized the introduction. We still discuss initialization in neural networks, because we think it is a related topic, but we make the relation to our work more clear (Lines 15-20). Finally, we show that a

random model with no further dynamical training can perform various working memory tasks, achieving longer memory than other models (Figure 5).

We did not think it was necessary to change the title, since our model is indeed “critically initialized”, regardless of how that might be related to ANN initialization, and only a model initialized in this way can capture the properties of the data. Additional training on the recurrent connections of a network would in fact change its spectral properties, making it less of a good model for our data. There are multiple ways to interpret this result:

(1) the random-initialized dynamics did not change because these mice were not yet trained in tasks that require persistent activity;
(2) during task performance, a different subset of the network may “turn on” and display different “learned” dynamics;
(3) no learning is in fact required on the recurrent connections; modifying input and readout connections is sufficient for most tasks (illustrated now in Figure 5, Lines 305-356).

We also added a discussion point to reflect these possibilities (Lines 399-409). If the reviewer considers that the title is still not ideal, we could switch to something like “*A critical network state for brainwide neural dynamics*”.

2. The abstract ends with a hypothesis that spontaneous activity reflects critical initialization optimized for learning time-dependent tasks. This claim lacks adequate support:

- What evidence supports this hypothesis? Which specific results in the paper substantiate these claims?

- If the authors believe their characterized dynamics benefit time-dependent task learning, direct evidence must be provided.

We made this more concrete by illustrating some of the computational properties of symmetric linear dynamics in the new Figure 5 (Lines 305-356). Consequently, we changed that last sentence in the abstract to:

“*These dynamics were useful for solving time-dependent tasks such as a zero-shot working memory task.*”

3. If critical initialization being optimal for learning represents an underlying theoretical assumption (rather than a hypothesis to be tested in this paper), it should be presented upfront with clear connections to existing literature. The paper's contribution relative to previous theoretical work requires better contextualization.

Unless the initialization connection can be substantially strengthened with direct evidence, I recommend removing it from the paper's central claims (including the title) and treating it as a discussion point. This would make the work more exploratory, which is acceptable, but would require better contextualization with prior related work and clearer questions.

We strengthened the connection to learning in Figure 5 and adjusted the motivations in the abstract and introduction. We overall added more references to echo state networks and liquid state machines to clarify the theoretical context, including a direct comparison in Figure 5.

Technical Comments:

1. The removal of train/test splits in SVCA analysis, justified through simulated data with added Gaussian noise (Fig S4), requires additional validation:

- Do results depend on noise amplitude or type?

We added two new noise conditions and the results strengthen the conclusion that SVCA2 is much less biased than SVCA or direct estimation for symmetric and partially symmetric simulations. Specifically, we added a noise condition meant to simulate the electrophysiology data (Poisson noise), and a second condition meant to simulate the 2p data (Poisson + calcium dynamics + shot noise + deconvolution) in Figure S4. In the same figure, we also tested the scaling with noise amplitude, and again found that SVCA2 is more robust than the previous method. Similarly for scaling with the number of neurons and timepoints, since reducing these parameters also acts as a sort of noise. We point out that SVCA had a consistently large bias positive bias even at large numbers of neurons and timepoints. SVCA2 slightly overestimated the power-law exponent at large numbers of neurons and timepoints, but this bias was much smaller, and nearly absent for the ephys-like Poisson simulation.

- The higher exponent estimations for noisy data shown in Fig S4 were somewhat expected: added noise may destroy higher principal components, leading eigenspectrum estimation to yield higher power-law exponents (reflecting lower-dimensional activity) for noisy versus noiseless data.

This is actually the reason why SVCA with train/test time splits overestimates the power-law exponent. When data is noisy, it becomes difficult to accurately identify the eigenvectors of the activity (N^2 parameters vs N for eigenvalues alone). As such, the variance projected onto train eigenvectors from the test data will be lower than expected, and even more so for eigenvectors with small eigenvalues, which are harder to identify. Thus, as the reviewer said, the data ends up *looking* low-dimensional with SVCA. However, this is not a property of the underlying shared dynamics, it is a property of the noise, and our goal is to factor out this bias.

Given the inherent noise in neural recordings, shouldn't SVCA account for this noise in estimations, perhaps through train/test splits? Without train/test splits, SVCA may overestimate dimensionality by failing to properly account for data noise. Can the authors elaborate more on the rationale behind their choice.

The single-neuron noise is mostly accounted for by having train/test splits in the neurons, which SVCA2 has. We say mostly because some amount will project into the covariance between train/test neurons, but that contamination scales like 1/T with the duration T, while the covariance of the signal between the two-neurons stays order 1. This is different when using the direct eigenvalue estimation method, because the diagonal noise variance stays order 1. It is also different when using standard SVCA, where the bias is due to poor estimation of eigenvectors (see previous comment), which is much harder to rectify by recording more neurons and timepoints because eigenvectors have so many more parameters.

- In general, sensitivity analysis showing how conclusions depend on SVCA approach choice would strengthen this methodological decision.

Thanks for the suggestion, we added more sensitivity analyses in Figure S4 and S6. In sum, these show that SVCA2 does a much better job than SVCA across a wide range of simulation conditions, and is generally more stable when changing analysis parameters in the real data too.

2. Two factors changed from previous work (Stringer et al., Science, 2019): GCaMP6s to GCaMP8s and removal of train/test splits in SVCA. Since the main claim involves V1 spontaneous activity matching symmetric random matrix eigenspectra, the individual contribution of each change to the lower power-law exponent estimation should be demonstrated separately; either via running the same analysis on the older data collected with GCaMP6s, or redoing the same analysis in this paper with train/test split.

Thanks for the suggestion, we added power-law exponent estimation step-by-step to go from 22Hz gcamp8s recordings to 3Hz gcamp6s recordings, including a comparison between microscopes (Figures S6e). The changes in power-law exponent are gradual. The only step that does not make a difference is changing the microscope, but every improvement in data quality changes the exponent. We think this justifies our original approach of trying to get the best data quality possible, and designing the best analysis method we can.

3. While bin size effects on exponent estimation are intuitive, several questions remain:

- How should the right bin size be determined? Which bin size would reflect the true dimensionality of activities? Wouldn't that require cross-validation?

We extended the analysis in Figure S3c to show that bin size does not substantially change the power-law exponent across bin sizes from 6 ms to 100 ms. Our 2p recordings are at 22Hz, so binning of ~45ms. On the ephys data, we added a new analysis with smaller bin sizes, and generally we find only small differences in power-law exponent across bin sizes (Figure S5de). These may be driven by the reduction in SNR at small bins, but we do not think they are large enough to be of concern.

- Can the linear dynamical system with random matrix interactions predict bin size effects on eigenspectrum estimation?

Yes, Figure S3c shows this in simulations. Bin size effects are negligible up to ~100 ms.

- Do predictions across different bin sizes consistently support the same conclusions regarding symmetric versus non-symmetric random matrix dynamics?

Bin size effects are negligible on bin sizes up to ~100ms for both symmetric and non-symmetric simulations (Figure S3c). The two types of simulations are also most distinguishable from each other at these small bin sizes, since at very large bin sizes the exponents become much more similar to each other (and correspond to very low-dimensional activity).

4. It would be helpful to demonstrate the sensitivity of the analysis to the number of recorded neurons. Power-law fits across different neuron numbers would establish minimum requirements for robust estimations.

Thanks, we added this analysis for both data and simulations. SVCA2 converges quickly with the number of neurons and timepoints, both in simulations and in real data (S4be, S6fgh).

5. Regarding the Neuropixels recordings:

- Which specific brain areas are included in the data?

We added this information in Lines 573-585. The detected neurons were in primary and higher order visual cortices, motor cortex, somatosensory cortex, the hippocampal formation, the caudate putamen, lateral septal cortex, superior colliculus, thalamus, and midbrain.

- While V1 and brain-wide activity show similar eigenspectral properties distinct from CA1, it seems unlikely that CA1 alone among all recorded areas exhibits different spectral properties.

Breaking down brain-wide recordings into functionally similar area subgroups could address this concern, though sufficient neuron numbers per area may be limiting (relating to comment #4).

We divided into 5 brain regions for quantification in the new Figure S5bc. This estimation is noisy and biased at low numbers of neurons. To compensate somewhat, we added recordings from PPC and from sensorimotor cortex, which show similar eigenvalue decay and DMD dynamics to the V1 and brainwide data (Figure 2). We cannot rule out that other brain areas stand out like CA1, but we did not find similar effects in any of the other areas we looked at.

Summary:

This work presents interesting findings on neural activity spectral properties, but the connection to network initialization requires substantial strengthening or removal from central claims. The technical analysis would benefit from additional methodological validation and clearer presentation of results across brain regions.

Referee #2 (Remarks to the Author):

1. Summary of the key results

The authors measure the scaling of the variance spectra of neurons in a mouse brain, which captures how quickly the variance of data falls off with the inclusion of an increasing number of principal component subspaces. The authors compare the found variance spectra with a random neural network model with fully random weights, and with a random neural network model with random weights that are "symmetrized", showing that the symmetrized network alone matches the variance scaling found widely across the mouse brain.

The authors further compare with a data-driven model: Dynamic Mode Decomposition (DMD). This model estimates the eigenvalues of the linearized dynamics, and the authors found that these eigenvalues have small imaginary part, consistent with the symmetric linear network model.

The authors conclude that the dynamics of the mouse brain mainly lack sequential/rotational structure, and are rather much more consistent with dynamics induced by symmetric connectivity profiles.

This is a good summary of our main results. Here is a summary of our revision:

- We added the additional analyses suggested below, which have strengthened the technical side of the paper.
- We divided the electrophysiology dataset into brain regions. These regions had approximately similar eigenvalue distributions to each other. However, the number of neurons per area became small, which introduced noise and bias into the estimation of the power-law exponent, diminishing the strength of our conclusions.
- To compensate, we recorded additional datasets from other brain regions accessible with large-scale 2p recordings.
- To get more active mice that can run while headfixed, we switched to a different genotype, expressed jGCaMP8s virally, and collected several more datasets.

- Finally, to round out the dataset that we will share publicly, we doubled the number of recordings in CA1, from 4 to 8. In total, we increased the number of datasets from 10 to 26.

Our conclusions stayed the same with the additional analyses and the new data.

2. Originality and significance

The work appears to be novel, including clarifying and extending existing theoretical work.

Thank you.

3. Data & methodology

The quality of data and validity of the collection methods appear to be high. One exception is that time binning in the case of the neuropixel data seems unnecessary and may impact the results. Is the time binning meant to capture a measure of membrane potentials or firing rates, and why? What is the downside of using the raw neuropixel data?

The results are presented in a clear way.

Some amount of binning is always needed in order to perform analyses like PCA or DMD. We added an analysis using smaller bins for ephys and found that the main results do not change qualitatively (Figure S5bc). We chose our "default" ephys time bin to be matched to the time resolution of the 2p data, thus allowing for easier integration and comparison between the two. Furthermore, binning allows for averaging out some of the Poisson-like variability in the data, which can be important for estimating the spectra accurately.

4. Appropriate use of statistics and treatment of uncertainties

Statistics appear to be properly treated.

5. Conclusions

We had a few comments to make on the results and conclusions:

5a. Using free probability theory applied to large matrices, we believe that the spectral properties of

$C = \int_0^\infty e^{At} e^{A^T t} dt$

(the integral solution to the continuous time Lyapunov equation) can be related to those of the symmetric random matrix $A + A^T$ as $N \to \infty$. Alternatively, one can argue that as $N \to \infty$, A and A^T become more commutative (the norm of their expected commutator shrinks to zero like 1/N). This reveals that the symmetric part of A governs the spectral scaling of C. Hence in the case of asymmetric A with the spectral radius approaching 1, one should find the same spectral scaling of C (even though the two scenarios have different asymmetric parts). Namely, in the idealized limit of $N \to \infty$ and time binning -> 0, the scaling in the case of Figure 1h and 1f should be the same.

Considering this, we recommend that the authors either:

(1) describe the flaws in our logic above if they exist, and perhaps clarify these points in the main text if appropriate

(2) if our logic above is correct, elucidate what details in the analysis result in different scalings of Figure 1h versus 1f such as time binning or finite N effects.

We think one of the steps in the argument is incorrect, specifically the commutator of A does not go to 0 as $N \to \infty$. For a non-symmetric matrix A, we have $A_{ij} \sim \mathcal{N}(0, 1/N)$ for a spectral norm of 1. When drawing random A with different sizes N, we empirically find the (Frobenius) norm of the commutator $||AA^T - AA^T||$ to be $\sim\sqrt{2N}$, while the norm of A itself is $\sim\sqrt{N}$. Since they are on the same scale, it means matrix A does not really become any closer to being commutative with larger N. And since A and A^T do not commute, then the matrix exponentials do not satisfy $e^{At} e^{A^T t} = e^{(A+A^T)t}$. There does appear to be an infinite series for Z in $e^Z = e^X e^Y$ called the Baker-Campbell-Hausdorff formula, but the first terms in the expansion are $Z = X + Y + ½ [X,Y]+\ldots$ with higher terms being higher-order commutators. But since the commutator – as well as higher-order ones actually – are the same size as X, Y, it does not seem immediately clear how to make progress.

5b. We believe the authors are somewhat vague on the precise brain areas that are recorded from using the neuropixel probes, and this description could be improved.

Thanks for the suggestion, we added a better description of this (Lines 573-585).

5c. Many sequential processes are known to occur in the rat brain, such as hippocampal replay and brain waves of various frequencies. The authors should discuss their results in relation to these known sequential processes, and if the time binning excludes these processes it should be justified.

Thanks for the suggestion. We analyzed multiple datasets with sequential structure from different studies and found that indeed some eigenvalues acquire imaginary parts (new Figure S8).

6. Suggested improvements

6a. The scaling exponents could easily be dependent on animal state. Since the animal is freely moving, this includes moments where the animal is still, and moments where the animal is moving. We recommend that the authors divide the dataset into sets of behaviors and analyze the scaling exponents and DMD results in these sets individually. A simple approach could be to simply divide the data into "moving" and "not moving" based on some threshold of detected movement.

Our initial data was mainly in the “not running” state, because the gcamp8 transgenic mice do not run well. To get data in the “running” state, we performed more recordings, including in a newly developed transgenic jGCaMP8s mouse, as well as in mice with a new combination of jGCaMP8s viruses which, to our knowledge, is the only combination to express homogeneously and brightly (Lines 456-460). We found a small difference in exponents between moving and not moving states (new Figure S7). The DMD analysis requires longer uninterrupted bouts of running, and thus we were only able to perform it in 3 mice, with no obvious difference between running and non-running eigenvalues.

6b. It is possible that the scalings reported from the neuropixel data are averages of different scalings in different brain regions. We believe it would be worth breaking the neuropixel probe

data into regions based on anatomical or functional considerations, and seeing if each of these regions yield the same scaling as the whole.

After breaking down the Neuropixel data into regions, we find relative homogeneity (Figure S5bc). The is some variation, but it is hard to disentangle from the noise induced by the much lower number of neurons. In particular, this analysis fails to identify the hippocampus as an area that has low power law exponents as a whole. However, it is likely that very few neurons from area CA1 are included in the ephys data, because these neurons fire extremely sparsely in spontaneous activity, and the orientation of the penetration is orthogonal to the stratum pyramidale, which is itself very thin (<100um).

6c. We think that a sanity check of the methods introduced by the authors could be helpful, perhaps in the supplementary materials. This would constitute testing their methods on either data where sequential behavior should be seen (such as motor cortex during walking) or on influential models of brain-driven sequential behavior, to show that the methods of the paper will give a positive result for asymmetry-driven dynamics in these basic cases.

Thanks. We now included analyses on six different datasets where rotational dynamics may be expected (Figure S8). We indeed find that DMD identifies several eigenvalues with imaginary parts. We present these results in the revision, but suggest caution in interpreting them, because the recordings have many fewer neurons, and also many fewer timepoints / trials compared to continuous uninterrupted spontaneous activity.

6d. As stated above in point (5c), the authors should clarify the choice of time bin for the neuropixel data and the potential consequences of this, such as being too coarse to capture brain waves or replay events.

Thanks, we show in Figure S5d (and S3c for simulations) that the choice of time bin does not make a big difference as long as it is less than 100ms. As for fast timescale oscillations, the symmetric model does not really predict oscillations (no modes have rotational eigenvalues), and we do not find them in the data, even when binning at very small timescales. Reconciling LFP oscillations with this data probably requires a different discussion, and it is not one we are well equipped to conduct in this particular manuscript with this particular kind of data.

7. References

This appears to be thorough.

8. Clarity and context

The manuscript is relatively clear, excepting points we make in some of the comments above.

Referee #3 (Remarks to the Author):

I co-reviewed this manuscript with one of the reviewers who provided the listed reports.

Thanks to the reviewers for their helpful comments, we have clarified their points by improving the diagrams in Figure 5 and adding a new summary panel in Figure S8.

Referee #1 (Remarks to the Author):

I thank the authors for their thorough response and for addressing the majority of my previous comments. I have several remaining questions regarding the working memory task, as detailed below:

The addition of the working memory (WM) task is a valuable step toward connecting the model's spectral properties to its functional performance. However, a few key points still require clarification:

- The structure of the WM task and the specific way models are implemented to solve it remain unclear. The current schematics do not sufficiently clarify the process; for example, the meaning of the dotted arrows in the diagrams is not defined. Please provide a more detailed walkthrough of the task implementation and ensure all visual symbols in the figures are explicitly explained.

Thanks for this feedback. We re-arranged the schematics in figure to make them more intuitive and easier to understand. We also added additional descriptions for the tasks in the main text.

- The manuscript would benefit from a more thorough justification of the performance differences between the models:

If the symmetric model's effectiveness stems from slower spectral decay—meaning more involvement of higher-order PCs—the authors should explain why this improves performance. Since higher-order PCs typically have faster timescales (as shown in figure 3), it is not immediately intuitive why they would enhance working memory capacity. The significant underperformance of the echo-state network is also not clearly explained. A more detailed mechanistic account is needed to help the reader understand why these specific spectral and architectural properties lead to such divergent results.

The main difference between the symmetric and non-symmetric linear models is due to rotations in the non-symmetric models. The rotations mean that a time-independent decoder cannot decode a stable representation over time. We added a couple of clarification sentences about this in the text. The echo state network has chaotic dynamics which are not robust to noise, and thus perform poorly. We also added this clarification in the text.

- The reasoning behind utilizing three different versions of the WM task should be made more explicit. It would be helpful to clarify whether each variant is intended to probe a specific facet of network capacity or a distinct computational bottleneck.

Thanks, we clarified this. Briefly, the first task is binary, so does not require capacity, but only timescales. The second task requires both capacity and timescales. The third task is equivalent to the second task with an additional storage mechanism (aligning inputs to eigenvectors of the network).

- There is a mismatch between the caption and the figure content. Fig 2 panel (b), for example, does not reflect the eight-probe Neuropixel recordings mentioned.

Thanks fixed.

Referee #2 (Remarks to the Author):

Than you for carefully addressing the issues. These additions appear to largely address our concerns, with some smaller points that we lay out below.

(5c and 6c) Thanks for these additions. We believe that a word of interpretation about the results in Figure S8, perhaps alongside a summary measure such as the rotation count, would be helpful to add to the manuscript.

Thanks, we have added a summary plot to Figure S8. We have also added another sentence in the text describing the results in Figure S8: "In other recordings where sequential activity is expected, for example in response to external, behaviorally-relevant cues, we found eigenvalues from DMD with significant rotational components (Figure S8). *We suspect that the rotational components observed in these recordings are at least in part related to the structure of the task and environment.*"

(6d) "Reconciling LFP oscillations with this data probably requires a different discussion, and it is not one we are well equipped to conduct in this particular manuscript with this particular kind of data."

Thank you for examining the effects of small time binning. We believe it would be helpful to include a brief discussion of possible reasons why the methods do not detect LFP oscillations, even when small time bins are used.

Thank you for the continuing discussion. We did a literature review and drafted a paragraph to be included in the discussion (see below). However, we then realized that there are too many assumptions implicit in such a discussion, many of which appear to be controversial or possibly invalidated based on our literature review. In the end, we decided not to include this brief discussion.

We are asked to discuss "why the methods do not detect LFP oscillations". We cannot detect LFP oscillations directly since we are analyzing spiking, not LFP. We would have to assume that there is at least some amount of spike-LFP coupling transferring the LFP oscillation to spikes. Second, we would have to assume that there are in fact oscillations in the LFP in these experiments, which we cannot confirm since we did not record the LFP. If we grant these two assumptions as true, we would then have to make an inference that our method does not detect the LFP oscillations, and discuss why that might be. Given that the assumptions are far from guaranteed (see paragraph below), we think it would be prudent not to give this subject a cursory treatment in the discussion. In the end, there simply may be no oscillations to detect, at least in the spiking activity of our data.

"*Of note is the lack of oscillatory dynamics in symmetric systems. While oscillatory dynamics are widely reported in LFP recordings, their coupling to spiking is well-understood only in a few cases, such as for the theta rhythm \cite{buzsaki2013memory}. Slower oscillations (<30Hz) are not typically present in neural population activity in awake animals \cite{Renart2010}. High-gamma oscillations are reported in cortex but have weak coupling to spiking \cite{watson2018temporal, dorman2023spike, martin2016phase}, and this coupling may be due to an analysis artifact \cite{scheffer2013high, ray2015challenges}. We did not find evidence of oscillations in the datasets we analyzed here.*"

Referee #3 (Remarks to the Author):

I co-reviewed this manuscript with one of the reviewers who provided the listed reports.
